# H-HQNN: Hybrid quantum-classical neural network with feature selection by heuristics to predict heart failure

**DOI:** 10.1371/journal.pone.0360391

**Published:** 2026-10-16

**Authors:** Neeraja Peddinti, M. Sowmya Vani

**Affiliations:** 1 Department of Computer Science, Mohan Babu University, Tirupati, Andhra Pradesh, India; 2 Department of Computer Applications, Mohan Babu University, Tirupati, Andhra Pradesh, India; Dr Shakuntala Misra National Rehabilitation University, INDIA

## Abstract

Cardiovascular diseases are the most dominant cause of mortality across the entire world, claiming 17.9 million lives every year. According to recent trends, the mortality rate as a result of heart failure continues to increase particularly in low- and middle-income areas which means that early diagnostic tools desperately need to be discovered. Conventional biomarkers like NT-proBNP, troponins and creatinine levels are useful, although their sensitivity and specificity are low, particularly in identifying heart failure onset before it is actually clinically apparent. We suggest addressing these issues by proposing the following Hybrid Quantum-Classical Neural Network with Heuristics-Based Feature Selection that can be used to predict the onset of heart failure: H-HQNN: A Hybrid Quantum-Classical Neural Network with Heuristics-Based Feature Selection. Given this, SMOTE is used to balance the clinical data and reduce class imbalance, whereas the most informative cardiac biomarkers and clinical features are selected using a two-stage process in which Recursive Feature Elimination (RFE) first prunes redundant attributes, followed by a dual-phase optimization where PSO refines the feature subset and Cuckoo Search (CS) enhances global exploration to avoid local minima, resulting in a compact and highly relevant feature set. These features are then represented in a variational quantum circuit (4 qubits) and are implemented as ZZFeatureMap, Pauli-Z gates, EfficientSU2, and RealAmplitudes, which are combined in a hybrid quantum-classical neural network. Performance was estimated using stratified 5-fold cross-validation on a single public dataset; external validation on independent cohorts has not yet been performed. The average accuracy of our model is 96.62% ± 3.45 with precision of 96.11, recall of 96.10, F1-score of 96.54 and AUC = 0.9604. The most efficient fold has 98.30% accuracy and AUC = 0.9872 with a confusion matrix of almost perfect balance in true positive and true negative rates. H-HQNN is more efficient and interpretable as it achieves better discriminative power, with fewer features, compared to classical baselines (e.g., Random Forests, deep neural networks). H-HQNN presents an opportunity toward novel methodology of identifying early heart failure by utilizing quantum-classical hybrid modeling and heuristic feature selection to ensure progress in preventative cardiology and therapeutic intervention in particular where advanced imaging studies or invasive measurements of biomarkers are not feasible.

## Introduction

Cardiovascular diseases (CVDs), of which heart failure is a major contributor, cause an estimated 17.9 million deaths worldwide each year [1]. It is commonly caused by some underlying factors like hypertension, diabetes, ischemic heart disease, and myocardial infarction that causes a decrease in the cardiac output and fluid retention [2]. The growing rates of HF especially in old age of people in the low and middle-income countries highlight the necessity of new methods of early detection [3]. Even though various biomarkers, including N-terminal pro-B-type natriuretic peptide (NT-proBNP) and troponins, are extensively utilized in the diagnosis process, their quality of prediction in the pre-symptomatic period is rather limited, often leading to late detection and the lack of therapeutic opportunities [4]. The complexity, and nonlinearity of the interplay between clinical, demographic, and laboratory factors that contribute to the progression of HF further underlines the necessity to model complex processes with sophisticated computational frameworks that are able to reveal a hidden diagnostic pattern in electronic health records that prepares the ground towards preventive cardiology.

In order to improve prognosis and minimise hospitalization, it is essential to diagnose HF in its early phases, as therapeutic measures can be used with a lot of success. Nevertheless, a multifactorial etiology of HF and lifestyle factors, including smoking, obesity and physical inactivity, make the prediction models complex. Although informative, conventional clinical scoring systems do not show nonlinear relationships between cardiac biomarkers, including “ejection fraction, serum creatinine, and sodium levels” [5]. The increase in cardiovascular incidents worldwide post-COVID-19 pandemic has only aggravated the necessity of predictive regimes incorporating biochemical, physiological and demographic measurements [6] in a single model of computation [3].

The use of machine learning (ML) and deep learning (DL) models to predict cardiac outcomes using heterogeneous clinical data has been on the rise. Random Forest, Gradient Boosting, and XGBoost ensemble methods have been shown to be reliable in the diagnostic prediction of coronary and heart failure risk [7], and neural networks and autoencoders have been used to predict temporal trends in electrocardiographic and echocardiographic data [8]. The development of deep learning has advanced the field further, and Deep Neural Networks (DNNs), automatically learn hierarchical feature representations, which might discover new prognostic signatures [9]. Deep neural networks are the most likely to capture nonlinearity, but they are also prone to overfitting because of small sample size and lack interpretability in medicine [8]. The traditional techniques involve manual feature engineering, which is time costly and may overlook underlined correlations between physiological characteristics such as serum creatinine, ejection fraction and sodium concentration biomarkers, which influence heart failure progression.

QML has evolved rapidly and has been able to implement quantum principles such as superposition and entanglement to enhance learning and representation. Other researchers such as [10] have proposed a quantum-enhanced heart failure prediction framework that combines a quantum CNN with Cuckoo Search-based feature qubit selection. Variational Quantum Circuits (VQCs) and Quantum Neural Networks (QNNs) have also been studied for transfer learning in hybrid classical-quantum networks [11] and for medical image classification [12], highlighting the applicability of QML to medical contexts. The Qiskit frameworks enable the execution of such hybrid systems with the help of available simulation and hardware backends. The literature of [13] and [14] indicates that Quantum Convolutional Neural Networks (QCNNs) and Quantum Support Vector Machines (QSVMs) can be used to solve small, imbalanced medical datasets. Moreover, meta-heuristic search engines like PSO and Cuckoo Search have proven to be successful in the optimization of clinical features subsets, which can be used to facilitate the combination of heuristic selection with quantum-enhanced learning [15].

Classical neural networks perform learning through interconnected neurons that process information using mathematical operations in Euclidean feature space. In contrast, hybrid quantum–classical neural networks integrate parameterized quantum circuits with classical optimization algorithms, enabling quantum operations such as superposition and entanglement to encode and transform feature representations before classical decision-making. Rather than replacing conventional neural networks, hybrid quantum–classical models complement classical computation by exploiting quantum feature mapping to capture complex nonlinear relationships that may be difficult to represent using purely classical architectures, particularly for small and high-dimensional datasets [16]. During training, the quantum circuit parameters and classical network parameters are jointly optimized using classical gradient-based optimization, resulting in an end-to-end learning framework that combines the expressive capability of quantum representations with the stability and scalability of classical deep learning.

Nevertheless, there are still a number of challenges to clinical adoption of Quantum Machine Learning (QML). The classical models of machine learning frequently suffer a performance bottleneck and are limited by the curse of dimensionality, forcing them to be explicitly and intensively engineered in terms of features [17]. Deep learning models offer higher representational power, but they are expensive in terms of data, overfit to smaller data with imbalanced samples [18], and not transparent because they render them less reliable in life-or-death medical tasks [19]. Recent QML frameworks are promising even though at an infantile phase. As an example of this, whereas [10] has shown the promise of quantum-enhanced heart failure prediction with Cuckoo Search-based feature selection, many hybrid quantum-classical models still omit heuristic feature selection such as PSO, which can improve interpretability and efficiency. In addition, the vast majority of implementations do not consider the hardware constraints of Noisy Intermediate-Scale Quantum (NISQ) devices, which lack qubits and are vulnerable to noise, and which require compact and optimized circuit designs [20]. Besides, numerous experiments are not thoroughly validated, e.g., k-fold cross-validation or ablation studies, which makes it hard to determine the specific value added by the quantum component. This means that current models be it classical, deep, or quantum still have a problem of scalability, interpretability, and clinical reliability and hence the need to have hybrid frameworks that integrate meta-heuristic optimization with quantum-enhanced learning to provide a robust clinical decision support system.

A heart failure prediction model, the Hybrid Heuristics-Driven Quantum–Classical Neural Network (H-HQNN), is developed in this study. The framework combines RFE, PSO and Cuckoo search to select heuristic features [10] with a 4-qubit variational quantum circuit implemented in Qiskit [21], where the ZZFeatureMap [22] is used to encode entangled features and the EfficientSU2 ansatz [21] is used as the compact variational learner. Important biomarkers including “creatinine phosphokinase, diabetes, ejection fraction, high blood pressure, serum creatinine, serum sodium, and sex” are encoded in four qubits and a complex relationship is achieved through quantum entanglements.

The quantum embedding will be combined with dense layers of classical computation, and the AdamW optimizer based on L1–L2 regularization is used to support convergence stability. In order to overcome current weaknesses in interpretability and a lack of balance in the data, the Synthetic Minority Oversampling Technique (SMOTE) [23] is used in the preprocessing of the dataset on the UCI Heart Failure Clinical Records [24]. After that, PSO [25] is made with Recursive Feature Elimination (RFE) [26] and enhanced by a Cuckoo Search [10] exploration stage to refine the PSO-generated subsets, to obtain a substantially small and discriminating set of clinical features, which are then used to reduce the input dimensionality to quantum encoding. The chosen features are embedded through ZZFeatureMap that induces quantum entanglement, then the EfficientSU2 variational processing variational. The model is trained as an end-to-end process with the input of quantum expectation values into classical neural layers leading to an efficient hybrid pipeline with optimisation under the AdamW algorithm to predict heart failure correctly and explainably.

The suggested H-HQNN model is tested on the dataset of the UCI Heart Failure Clinical Records where the 5-fold cross-validation is utilized to provide the statistical soundness. The hybrid quantum -classical architecture can be compared with classical ML baselines, and it is estimated to be superior, achieving the accuracy of 96.62% ± 3.45, precision of 96.11, recall of 96.10, F1-score of 96.54, and AUC of 0.9604. The findings suggest that hybrid quantum learning has the capacity to be applied in clinical diagnostics, especially to high-impact diseases where interpretability, generalization, and computational efficiency are important. The hybrid combination of PSO feature optimization and quantum encoding improves the discriminative representation in small, unbalanced datasets.

The broad findings of this paper are summarized to be as follows:

1. Heuristics-based Feature Optimization: PSO is applied to select the most discriminative biomarkers, eliminating redundancy and enhancing the interpretability of the model.
2. Quantum-Enhanced Representation: A 4-qubit Qiskit circuit based on ZZFeatureMap with EfficientSU2 allows the expressive encoding of clinical data using quantum features.
3. The H-HQNN model combines quantum and classical layers to support heart-failure survival binary classification.
4. Thorough Assessment: Five-fold stratified cross-validation shows consistent performance across folds on the UCI Heart Failure Clinical Records dataset; generalization to independent cohorts remains to be evaluated.

Organization of the remaining part of the paper: Section 2 will discuss the predictions of heart failure and quantum-enabled systems. Section 3 is describing the model architecture and methods. Section 4 has experimental and comparative findings. Section 5 deals with the performance and clinical consequences, and Section 6 concludes with the future research.

## Literature review

The studies on ML and DL heart failure prediction systems have enhanced accuracy and reliability over the decades all over the globe. SVMs, DTs, and RFs were average but could not cope with large dimensional data and non-linear nature of medical imaging data [27]. Classical models perform well but rely heavily on manual feature engineering and fail to capture complex multimodal patterns. QML overcomes these limits by exploiting superposition and entanglement to process high-dimensional medical data efficiently. Emerging hybrid quantum-classical models thus offer powerful path toward more accurate and scalable cardiovascular diagnostics. They developed an optimized quantum SVM (OQSVM) and a hybrid quantum multi-layer perceptron (HQMLP) for cardiovascular classification, reaching 94% and 93% accuracy respectively [28]. Heart disease prediction using instance-based quantum ML and DL [13] on the Cleveland heart disease dataset optimized qubits based on feature selection and improved prediction performance. The proposed quantum DL and quantum ML models achieved accuracies of 98 percent and 83.6 percent respectively, demonstrating the effectiveness of quantum learning for early disease detection.

The recent developments in medical data analytics created awareness of the growing potential of hybrid and quantum-inspired algorithms in cardiovascular diseases prediction [15]. suggested the Multi-Layer Perceptron, which was trained using PSO to improve the accuracy of diagnostic on clinical data. Their model attained an accuracy of 94.8 percent with the improvement of the convergence and interpretability of the feature weights, which demonstrated the capability of the meta-heuristic in improving the generalization. Nevertheless, the research was based solely on classical education, and quantum encoding or qubit-based optimization was not integrated, which created an opportunity of hybrid architectures.

Troponin, CRP, and lipoprotein(a) assays are examined internationally to enhance prediction models. Calcium variables like CAC scores increase cardiac risk prediction [29]. Heart disease kills most worldwide. Early CVD identification minimizes heart attacks and accelerates recovery. CVD angiography is expensive and hazardous. Current CVD diagnostic tools’ sluggish convergence rates need several rounds to discover the optimal alternative. So, detection rates fall. Use QPSO and SVM to forecast heart disease susceptibility. Preprocessing begins with numericization and nominal scaling. The QPSO-SVM model enhances SVM optimization using Quantum Particle Swarm Optimization (QPSO) with a self-adaptive threshold to refine parameter tuning and prevent convergence to local minima. By efficiently exploring the solution space, the approach achieved 96.31% accuracy, outperforming other models on the Cleveland heart disease dataset, with strong metrics including F1-score (0.95%), sensitivity (96.13%), specificity (93.56%), and precision (94.23%) [30]. Similarly, an Aquila-optimized hybrid QNN–LSTM model with feature selection reached 96.69% accuracy for cardiovascular disease prediction, demonstrating the effectiveness of meta-heuristic optimization in hybrid quantum architectures [31].

[32] developed a PSO-tuned stacked sparse autoencoder for cardiac risk assessment that achieved 96.2% accuracy on the Cleveland Heart Disease dataset. The model demonstrated PSO’s effectiveness in feature dimensionality reduction and convergence stability. Despite strong results, the computational cost was high, and the study did not explore robustness under data imbalance an aspect addressed in later hybrid designs using SMOTE and PSO together, as in the present work.

Quantum-enabled architectures are increasingly being investigated for cardiovascular diagnostics [14]. proposed a QML-based heart-failure detection framework that outperformed classical models, reaching 93.7% accuracy and 0.94 AUC using hybrid variational quantum circuits implemented in Qiskit. The work validated the feasibility of encoding heart-failure biomarkers as quantum states but lacked a systematic ablation across ansatz types or feature-selection methods. On the same note [33], revealed a quantum-enhanced machine-learning (QuEML) pipeline in diagnosing heart diseases with an accuracy of 91.5 percent, using the circuits of RealAmplitudes. Even though performance improvements over CNN and SVM baselines were small (around 0.6 percent), this study enhanced reproducibility and opened the door to Qiskit-based hybrid medical model.

A heart failure survival prediction model [34] using Gradient Boosting Machine (GBM) and Adaptive Inertia Weight Particle Swarm Optimization (AIW-PSO) achieved a test accuracy of 94 percent on a Kaggle dataset of 299 patients, demonstrating the effectiveness of adaptive hyperparameter optimization for clinical prediction. Nevertheless, the study was limited by its small dataset size and lack of external validation, which may affect its generalization in real-world clinical settings.

The healthcare potential of quantum computing has been put into perspective using several surveys [35]. gave a list of proposed quantum hardware and algorithm uses in medicine, noting its potential to be used in genomic, radiologic, and cardiovascular applications but pointing out that reproducibility and scaling to circuit depth are issues [36]. has discussed Support Vector Machines (SVMs) in medical AI, with results of 90–95% accuracy in diagnostic data and that such classical baselines continue to be crucial comparators of hybrid QML [37]. introduced a digital-analog Quantum Convolutional Neural Network (QCNN) with 97.1% image accuracy, with 40 times fewer parameters, which demonstrated the efficiency of small ansatz designs that can be made medical specific.

Improvements to the Quantum k -Nearest Neighbor (QkNN) algorithm were described in [38] through a polar-distance metric, where an average accuracy of 96.5% is obtained on benchmark sets and the consistency of classification is improved. Although this is not a cardiac-specific experiment, this article demonstrated that quantum circuits of small size were capable of performing better than classical neighbors when incorporated into expressive Hilbert spaces. A quantum framework of Healthcare 4.0 was proposed by [39] with a focus on throughput and scalability of high-volume biomedical pipelines. Their contribution highlighted the role played by hybrid architectures in enabling real-time clinical analytics. Lastly [40], proposed a hybrid framework combining classical and quantum-inspired techniques, in which a quantum genetic algorithm selected features and a quantum particle swarm optimizer tuned a quantum SVM; on a combined heart disease dataset of 918 samples it reached 97.83% accuracy, compared with 94.02% for its classical counterpart. The study addressed general heart disease rather than heart failure records.

PSO algorithms performed worse than MBPSO. For cardiac diagnosis, use hybrid PSO-SVM [41]. PSO automatically reduced feature count to improve SVM classifier accuracy. Globally, an estimated 17.9 million people die from cardiovascular diseases each year [1]. Complex, high-dimensional data, disparities in class interpretability, and short dataset performance challenge traditional algorithmic heart disease prediction algorithms. Not all hybrid models effectively integrate Quantum Machine Learning (QML) due to challenges in scalability and generalization. The Kolmogorov–Arnold Classical–Quantum Dual-Channel Neural Network (KACQ-DCNN) addresses this by approximating continuous equations through Kolmogorov–Arnold Networks (KANs) with learnable univariate activation functions. Using a 4-qubit, single-layer design, KACQ-DCNN achieved 92.03% accuracy and 92.00% average precision, recall, and F1-score, outperforming 37 comparable models [42]. It also surpassed nine top baselines in single-tailed paired t-tests with an ROC-AUC of 94.77% (αadjusted = 0.0056). Ablation studies confirmed the benefit of quantum-classical interaction, while LIME and SHAP enhanced model interpretability and clinical confidence [42].]. The related studies are summarized in Table 1., highlighting the datasets, methodologies, and performance of quantum and hybrid machine learning approaches for heart disease prediction.

**Table 1:** Related Works on Quantum and Hybrid Machine Learning for Heart Disease Prediction.

Ref No	Method Used	Measures	Key Outcomes	Research Gaps
[13]	Quantum ML and DL	Quantum DL is 98 and Quantum ML is 83.6	Demonstrated potential in early detection of heart related issues.	Integration with existing healthcare systems
[14]	Quantum Machine Learning (QML) Framework	Accuracy: 93.7%, AUC: 0.94	Outperformed classical baselines using Qiskit-based quantum circuits	No ablation across ansatz or feature selection strategies
[15]	MLP-PSO Hybrid Model	Accuracy: 94.8%, Precision: 94.2%	Improved diagnostic accuracy via PSO-based feature weighting	No integration with quantum circuits; lacks scalability analysis
[27]	Ensemble methods (Random Forest)	Accuracy: 90.16%	Outperformed models like SVM and XGBoost in diagnostic applications	Overfitting, data imbalance
[28]	Optimized Quantum SVM (OQSVM) and Hybrid Quantum MLP (HQMLP)	Accuracy: 94% (OQSVM), 93% (HQMLP)	Predicted ischemic heart disease effectively	Quantum hardware limitations, scalability
[30]	QPSO-SVM	Accuracy: 96.31%, Sensitivity: 96.13%	Predicted heart disease better than competitors	Parameter tweaking optimization for local minima
[31]	Aquila-optimized feature selection with QNN-LSTM	Accuracy: 96.69%	Enhanced predictive accuracy for cardiovascular diseases	Broader application in other areas of healthcare
[32]	PSO-Tuned Deep Autoencoder	Accuracy: 96.2%, F1-score: 95.9%	Enhanced feature dimensionality reduction and convergence	High computation cost; imbalance not addressed
[33]	Quantum-Enhanced Machine Learning (QuEML)	Accuracy: 91.5%, F1-score: 90.8%	Introduced RealAmplitudes ansatz for healthcare prediction	Marginal improvement over CNN/SVM; small dataset
[34]	GBM + AIW-PSO, SMOTE, feature selection	299 patients, AIC, BIC, accuracy	94% test accuracy, improved prediction performance	Small dataset, no external validation, limited generalization
[35]	Quantum Computing in Medicine (Review)	Theoretical Coverage	Discussed quantum potential in genomics and cardiology	Reproducibility and hardware scalability not addressed
[36]	Support Vector Machine (SVM) in Medical AI	Accuracy: 90–95%	Classical baseline for clinical classification tasks	Limited adaptability to high-dimensional data
[37]	Digital-Analog QCNN	Accuracy: 97.1%, Parameters ↓40%	Achieved strong accuracy with reduced circuit depth	No validation on structured tabular data
[38]	Enhanced Quantum K-Nearest Neighbor (QkNN)	Accuracy: 96.5%, AUC: 0.95	Improved classification consistency via polar-distance metric	Not applied to clinical biomarkers
[39]	Quantum-Enabled Healthcare 4.0 Framework	Throughput, Latency	Scalable architecture for large-scale medical data	Lack of disease-specific evaluation
[40]	QGA-QPSO-QSVM (quantum-inspired hybrid)	Accuracy: 97.83% (classical CGA-CPSO-CSVM: 94.02%)	Quantum-inspired feature selection and QSVM outperformed the classical counterpart	General heart disease data (918 samples), not heart failure; classical simulators only
[42]	KACQ-DCNN	Accuracy: 92.03%, ROC-AUC: 94.77%	Trusted cardiovascular diagnosis platform	Integrating healthcare practices, developing

Collectively, these studies reveal consistent themes: (i) meta-heuristics such as PSO and CSO substantially improve feature selection and convergence; (ii) quantum encoding via feature maps like ZZFeatureMap and EfficientSU2 enhances classification accuracy on small, high-dimensional datasets; and (iii) few works have yet combined both paradigms within a unified hybrid neural architecture that integrates regularization, cross-validation, and statistical significance testing. The deep ablation testing, feature-selection optimization and robust cross-validation, which are crucial in clinical applicability.

## Proposed methodology

The entire process of the proposed Hybrid Quantum–Classical Neural Network (H-HQNN) Heart Failure Prediction is shown in Fig 1. It starts with the Heart Failure Prediction Dataset, which is subjected to the data preprocessing stage, including handling missing values, normalizing the attributes, and encoding the categorical variables. Data augmentation is then applied to increase dataset diversity and address the issue of class imbalance. After preprocessing, Recursive Feature Elimination (RFE) is used to extract and rank the most informative attributes, which are subsequently refined through a two-stage heuristic optimization in which PSO, combined with Random Forest (RF), identifies the strongest candidate subset (q = 4), and Cuckoo Search further enhances global exploration to avoid local minima and ensure a compact, highly discriminative feature set. These optimized features are then encoded into quantum states through a feature-to-qubit mapping process.

**Fig 1:**
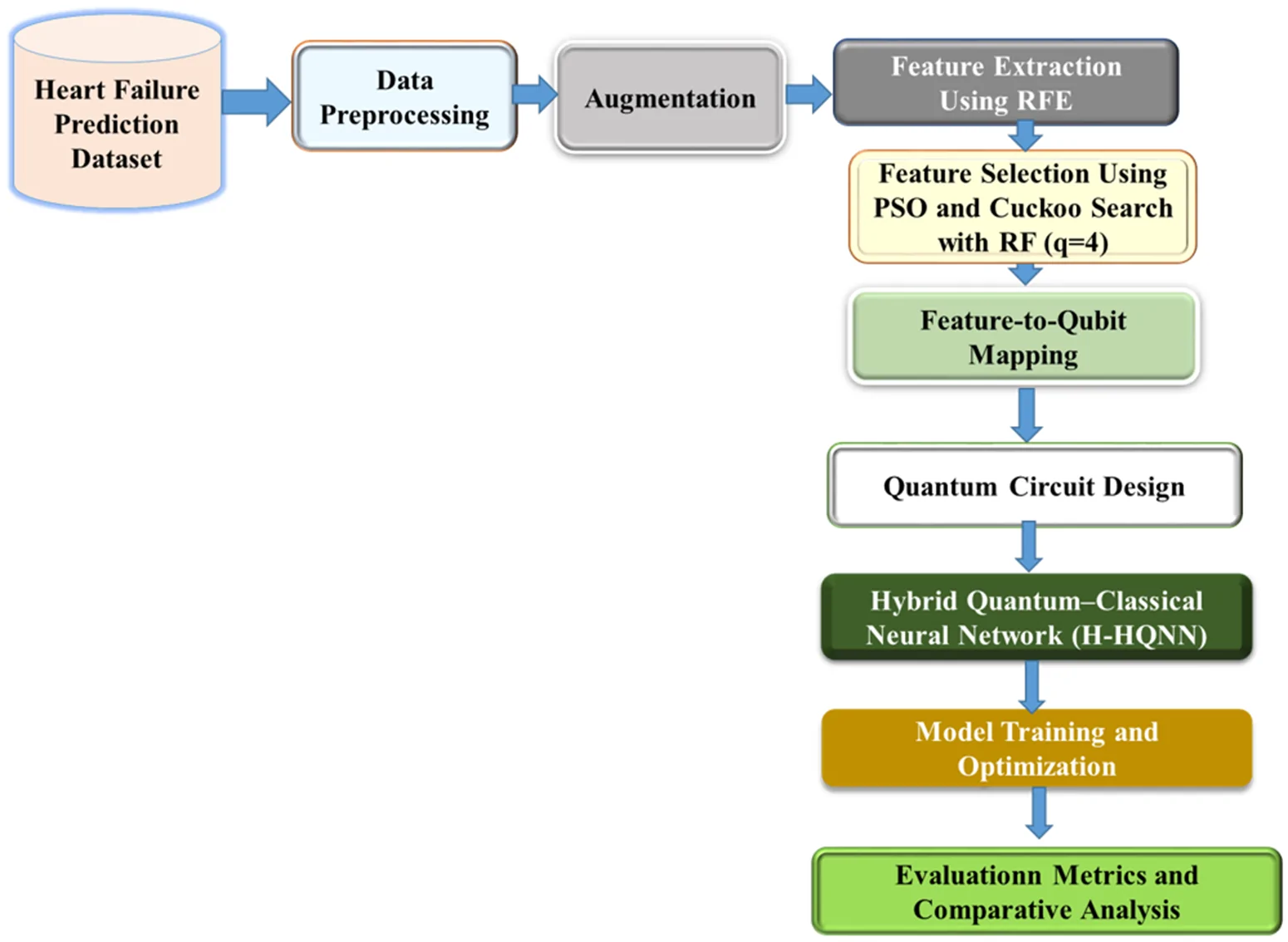
Proposed Methodology.

This is then followed by a quantum circuit that is developed to record quantum correlations and entanglement between qubits, which forms the basis of hybrid computation. H-HQNN model combines both the classical layers in deep learning and the quantum layers to take advantage of quantum parallelism and neural representation learning. Parameters are adjusted in the process of model training and optimization to reach convergence at a small loss and a high accuracy. Lastly, the measurement metrics and the comparison are conducted to compare the recital of the proposed model with the current approaches to show its strength, readability and high predictive power in the diagnosis of heart failure.

In the proposed H-HQNN framework, the quantum circuit functions as a nonlinear feature transformation module, whereas the classical neural network performs parameter optimization and final prediction. This hybrid design combines the representational advantages of quantum feature encoding with the efficient optimization capabilities of classical learning algorithms.

### Data preprocessing and augmentation

The dataset of the UCI Heart Failure Clinical Records has 13 clinical and demographic variables. The absent cases were dropped and the discrete variables were coded to binary variables. To overcome the imbalance of data (203 survivors, 96 deaths), the SMOTE technique was used to produce a balanced dataset, which consisted of 406 samples (203 survivors and 203 deaths). Continuous features were normalized to the range [0,π] using Min–Max scaling as:


$$\begin{array}{c} x_{i}^{scaled}=\;\pi .\;\frac{x_{i}-\text{min}(x)}{\text{max}(x)-\text{min}(x)} \end{array}$$ (1)

Gaussian noise augmentation was used on continuous features to increase generalization. If $x_{i}$ denotes the original feature value and $\epsilon \sim \;N(\text{0},\;\sigma^{\text{2}})$, then:


$$\begin{array}{c} x_{i}^{aug}=x_{i}+\epsilon ,\;\sigma =\text{0}.\text{0}\text{1} \end{array}$$ (2)

The preprocessing pipeline maintains an equal representation of the classes and resists the small perturbations in the clinical biomarkers.

### Feature extraction and selection

RFE by ranking the predictors by the importance of the Random Forest was initially used to eliminate irrelevant predictors to refine the feature space. Based on the subset of reduced data, PSO was utilized to determine the best set of biomarkers to use in quantum encoding.

Each particle represents a binary mask m∈{0,1}^d^, with exactly k = 4 active features. The objective function maximizes classification accuracy while minimizing redundancy:


$$\begin{array}{c} J(m)=\;\lambda \;.\;Acc(F⊙m)-(\text{1}-\lambda ).\;Redundancy(m) \end{array}$$ (3)

where F is the feature matrix, ⊙ denotes element-wise multiplication, and Redundancy(m) is measured via mutual information.


$$\begin{array}{c} v_{i,d}(t+\text{1})=w.v_{i,d}(t)+c_{\text{1}}.r_{\text{1}}.({pbest}_{i,d}-x_{i,d}(t)+c_{\text{2}}.r_{\text{2}}.({gbest}_{d}-x_{i,d}(t) \end{array}$$ (4)

$$\begin{array}{c} x_{i,d}(t+\text{1})=x_{i,d}(t)+v_{i,d}(t+\text{1}) \end{array}$$ (5)

After PSO identifies a high-quality candidate subset, Cuckoo Search is used as a global refinement stage. Its Lévy-flight-based exploration helps escape local minima and perturbs the PSO-selected feature masks to discover more globally optimal solutions. The nests with lower fitness values are replaced, ensuring that the final subset exhibits both high predictive power and minimal redundancy.

The final heuristic selection produced the discriminative biomarkers such as “demographic, high blood pressure, ejection fraction, serum creatinine, serum sodium, creatinine phosphokinase”. These selected features were then mapped to a 4-dimensional latent quantum space through a dense layer prior to feature-to-qubit projection.

### Feature-to-Qubit mapping

The selected biomarker set $\;F_{PSO}\in R^{n}$ is projected to a d dimensional latent vector by some fully connected projection:


$$\begin{array}{c} z=W.\;F_{PSO}+b,\;z\in \;R^{d} \end{array}$$ (6)

The z elements serve as a rotation parameter of each of the qubits and provide compatibility between classical properties and quantum encoding.

### Quantum feature encoding and variational circuit

Each reduced feature vector derived through PSO + CS,


$$\begin{array}{c} F_{pSO}=[x_{1},x_{2},x_{3},x_{4}]\in \mathbb{R}4 \end{array}$$ (7)

is designed as a four-qubit quantum system in Qiskit based on feature mapping and variational circuit design. The quantum representation encodes the clinical biomarkers into entangled states that describe the nonlinear interdependences between the clinical biomarkers that are critical in heart-failure prognosis.

#### ZZFeatureMap encoding.

The ZZFeatureMap which is part of the Qiskit algorithm accepts classical feature inputs and converts them to quantum correlations using parameterized Pauli-Z rotations and controlled entangling operations. The phase relationships of the interactions between features are coded in pairs.


$$\begin{array}{c} U_{ZZ}(x)=\;\prod_{i<j}\text{exp}(i.\pi x_{i}x_{j}Z_{i}Z_{j}) \end{array}$$ (8)

Following initialization of all qubits with Hadamard gates, the resulting encoded state is


$$\begin{array}{c} {\left|\;\psi_{ZZ}\rangle =U_{ZZ}(x)H^{⨂\text{4}}\right|\;\text{0}\rangle }^{⨂\text{4}} \end{array}$$ (9)

This representation embeds second-order dependencies among biomarkers for example, the combined impact of diabetes and hypertension enhancing the separability of high-risk and low-risk cardiac profiles in Hilbert space.

#### RealAmplitudes ansatz.

The RealAmplitudes variational ansatz constructs a layered sequence of parameterized RY rotations followed by entangling CNOT operations. For n = 4 qubits, the general unitary is expressed as


$$\begin{array}{c} U_{RA}(\theta )=\;\prod_{l=\text{1}}^{L}\prod_{q=\text{1}}^{\text{4}}R_{Y}\left(\theta_{l,q}\right).CX \end{array}$$ (10)

Each $\theta_{i}$ is a is a scaled version of the input feature $z_{i}$:


$$\begin{array}{c} \theta_{i}=\;\alpha .z_{i} \end{array}$$ (11)

The single-qubit rotation matrix RY(θi)) is defined as:


RY(θi)= [cos(θi2)sin(θi2) −sin(θi2)~~cos(θi2)] (12)

Stacking L layers of this form increases circuit expressiveness while maintaining trainability:


$$\begin{array}{c} U_{RA}^{\left(L\right)}=\;{\left(R_{Y}.CX\right)}^{L} \end{array}$$ (13)

The RealAmplitudes configuration enables smooth parameter optimization and robust learning of survival patterns in the clinical dataset.

#### EfficientSU2 – Compact entangling ansatz.

The EfficientSU2 variational form in Qiskit provides a compact, hardware-efficient alternative that combines RY and RZ rotations with entangling layers:


$$\begin{array}{c} U_{ESU\text{2}}(\theta )=\;\prod_{l=\text{1}}^{L}\prod_{q=\text{1}}^{\text{4}}\left[R_{Y}\left(\theta_{l,q}^{\left(y\right)}\right)R_{Z}\left(\theta_{l,q}^{\left(Z\right)}\right)\right].Entangle(q) \end{array}$$ (14)

This structure balances expressiveness and circuit depth, making it suitable for NISQ devices. The EfficientSU2 ansatz was experimentally tested alongside RealAmplitudes within the H-HQNN pipeline to evaluate convergence stability and predictive robustness for heart-failure risk prediction.

#### Quantum circuit construction for H-HQNN.

The last quantum feature encoding in the H-HQNN scheme consists of incorporating the four chosen biomarkers to a 4-qubit Qiskit circuit consisting of a feature map and a variational ansatz. The normalized and PSO + CS-optimized features are coded in the ZZFeatureMap into entangled nonlinear quantum states and represent nonlinear relationships between serum creatinine, ejection fraction, and blood pressure as cardiac biomarkers:


$$\begin{array}{c} U_{feature}=ZZFeatureMap(F_{PSO}) \end{array}$$ (15)

$$\begin{array}{c} U_{ansatz(\theta )}=EfficientSU\text{2}(\theta ) \end{array}$$ (16)

The combined quantum circuit


$$\begin{array}{c} U_{qlayer}=U_{ansatz(\theta )}.\;U_{feature} \end{array}$$ (17)

encodes the selected biomarkers into a 4-qubit quantum state, from which expectation values of Pauli-Z operators are measured. These measured observables form a dense embedding vector representing quantum-enhanced feature correlations.

The resulting measurement vector is concatenated with classical activations in the hybrid neural architecture and propagated through dense layers for binary classification of heart-failure survival vs. death events. Circuit parameters are optimized via gradient-based AdamW learning, enabling seamless integration between quantum encodings and classical backpropagation. This unified construction allows the H-HQNN model to exploit both the quantum circuit’s entanglement expressivity and the neural network’s nonlinear discriminative capability for robust cardiovascular risk prediction.

### Hybrid quantum–Classical neural network (H-HQNN)

The H-HQNN integrates quantum measurements (⟨Z⟩ expectation values) into a classical deep learning pipeline for end-to-end optimization. The PSO + CS-selected features are first processed through batch normalization and nonlinear activation before entering the quantum layer, which captures complex entangled relationships. These quantum outputs are interred with classical dense layers, regularized using dropout, and then through a softmax layer to give the final heart failure prediction probabilities.


$$\begin{array}{c} h_{\text{1}}=LeakyReLU(BN\left(W_{\text{1}}F_{PSO}+b_{\text{1}}\right)) \end{array}$$ (18)

$$\begin{array}{c} q=Qlayer\left(h_{\text{1}}\right), \end{array}$$ (19)

$$\begin{array}{c} h_{\text{2}}=dropout(LeakyReLU\left(BN\left(W_{\text{2}q}+b_{\text{2}}\right)\right)) \end{array}$$ (20)

$$\begin{array}{c} \hat{y}=softmax(W_{\text{3}}h_{\text{2}}+b_{\text{3}}) \end{array}$$ (21)

### Model training and optimization

Training was performed using AdamW optimizer with learning rate 1 × 10 − 4. To reduce overfitting, L1 (λ = 1e-5) and L2 (λ = 1e-4) regularization were employed along with dropout (0.1). The binary cross-entropy loss was minimized:


$$\begin{array}{c} L(\theta )=\;-\;\frac{\text{1}}{m}\sum_{i=\text{1}}^{m}\left[y_{i}log{\hat{y}}_{i}+\left(\text{1}-y_{i}\right)\text{log}(\text{1}-{\hat{y}}_{i})\right] \end{array}$$ (22)

A 5-fold stratified cross-validation ensured fair evaluation across balanced splits. Early stopping with patience of 20 epochs prevented overfitting.

#### Model architecture.

As illustrated in Fig 2., the H-HQNN model architecture comprises three main stages integrating heuristic optimization with hybrid quantum–classical learning. Initially, the balanced dataset undergoes feature refinement through RFE–PSO–CS, generating a compact and discriminative feature vector that forms the network input. This vector is processed by a series of classical layers (fc1 → bn1 → act1 → fc2) for normalization and nonlinear transformation before being mapped into a 4-qubit quantum circuit constructed using a FeatureMap and Variational Ansatz. The quantum layer encodes the optimized clinical features into entangled states, capturing nonlinear interdependencies among biomarkers. The resulting quantum expectation values are then concatenated with classical activations and propagated through additional dense layers (fc3 → bn3 → act3 → dropout → softmax) to perform the final binary classification of heart failure outcomes, combining quantum expressivity with classical learning for heart failure outcome prediction.

**Fig 2:**
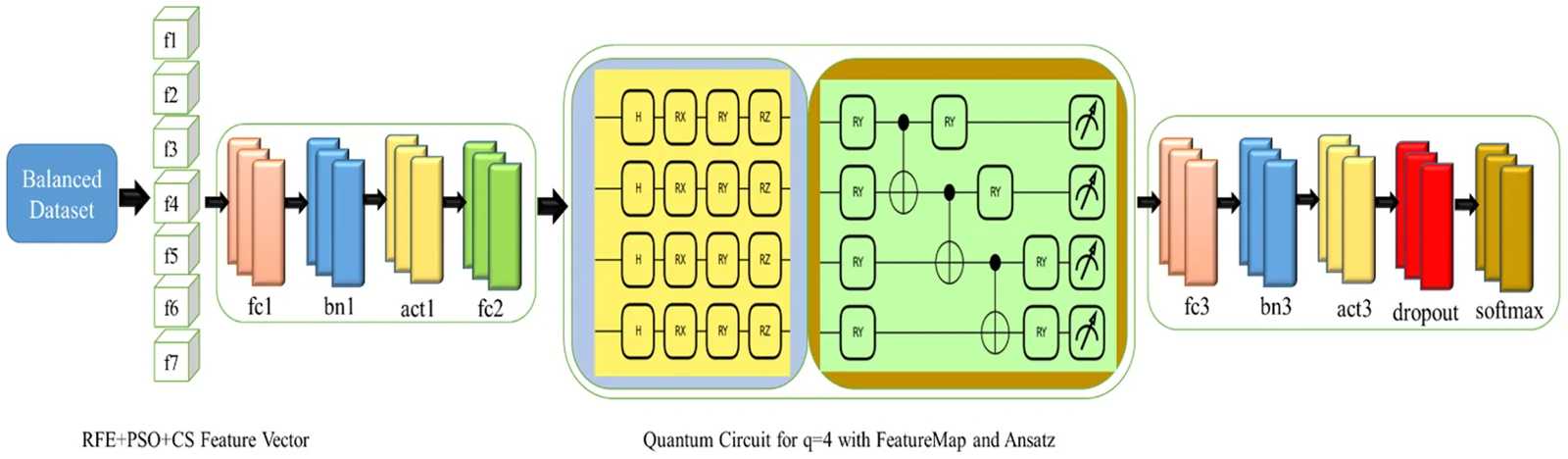
H-HQNN Model Architecture.

### Algorithm H-HQNN model workflow

The proposed H-HQNN algorithm begins with data preprocessing and RFE–PSO–CS-RF feature selection to extract the seven most discriminative biomarkers. These selected features are mapped into a 4-qubit quantum circuit comprising a FeatureMap and Variational Ansatz, enabling the modeling of nonlinear entangled relationships among cardiac biomarkers. The quantum outputs (⟨Z⟩ expectation values) are then integrated with classical dense layers for hybrid training, culminating in a binary classification of heart-failure outcomes with enhanced interpretability and predictive precision.

Algorithm H-HQNN (Hybrid Heuristics–Driven Quantum–Classical Neural Network)

Input:

  D_raw  ← raw heart-failure dataset (features X, label y)

  k_folds ← 5

  q ← 4 // number of qubits

  η ← 1e-4 // AdamW learning rate

  λ1, λ2 ← 1e-5, 1e-4 // L1/L2 regularization

  patience ← 20 // early stopping

  batch_size ← 16

Output:

Trained model θ*, fold-wise metrics (Accuracy, Precision, Recall, F1, AUC)

Step 1: PREPROCESSING

 D ← remove_missing(D_raw)

 X_num_, X_cat_ ← split_numeric_categorical(X)

 X_cat_ ← encode_categorical(X_cat) // one-hot or binary

 X_num_ ← minmax_scale_to_[0, π](X_num)

 X_num_ ← X_num_ + Normal(0, σ=0.01) // Gaussian noise augmentation

 D_bal_ ← SMOTE(D, target=y) // balance classes

Step 2: FEATURE SELECTION (RFE + PSO)

 F0 ← RFE(RandomForest, D_bal.X_, D_bal.y_) // coarse pruning

 M1 ← PSO_optimize_mask(F0, k=4) // binary mask of 4 biomarkers

 m*← CS_optimize_mask(M1, k=4)

 X_sel_ ← F0[:, m*] // selected features

 z ← DenseProjection(X_sel_ → ℝ^^^q) // fc1 → bn1 → act1 → fc2

Step 3: QUANTUM LAYER (q = 4)

 // Build feature map and ansatz

 Φ ← ZZFeatureMap(num_qubits=q) // encodes z into entangled state

 A ← EfficientSU2(num_qubits=q, layers=L) // variational circuit

 Qlayer(z; ϕ, α) = ExpectationZ(A(Φ(z))) // ⟨Z⟩ on each qubit → ℝ^^^q

Step 4: CLASSICAL HEAD

 h1 = LeakyReLU(BN(fc1(X_sel_)))

 qv = Qlayer(DenseProjection(h1 → ℝ^^^q))

 h2 = Dropout(LeakyReLU(BN(fc2(qv))))

 ŷ = Softmax(fc3(h2))

Step 5: TRAINING (k-fold CV)

 for fold in 1..k_folds do

  (train, val) ← stratified_split(D_bal, fold)

  Initialize θ = {weights of fc’s, BN, and quantum params α}

  best_val_loss ← +∞ ; no_improve ← 0

  while not converged do

   for (xb, yb) in mini_batches(train, batch_size) do

    // forward

    h1 = LeakyReLU(BN(fc1(xb; λ1, λ2)))

    qv = Qlayer(DenseProjection(h1 → ℝ^q^; λ1, λ2))

    h2 = Dropout(LeakyReLU(BN(fc2(qv; λ1, λ2))))

    p = Softmax(fc3(h2; λ1, λ2))

    // loss (binary cross-entropy + L1/L2 penalties)

    L = BCE(p, yb) + λ1*L1_norm(θ) + λ2*L2_norm(θ)

    // backward + update with AdamW(η)

    θ ← AdamW_step(θ, ∇θ L)

   end for

   // validation + early stopping

   L_val_ = evaluate_loss(val, θ)

   if L_val_ < best_val_loss then

    best_val_loss ← L_val_; θ* ← θ; no_improve ← 0

   else

    no_improve ← no_improve + 1

    if no_improve ≥ patience then break

   end if

  end while

  // metrics on fold

  Metrics[fold] ← compute_metrics(val, θ*) // Acc, Prec, Rec, F1, AUC, CM

 end for

 return θ*, summarize(Metrics)

## Results and discussion

QML is realized by the creation of a Hybrid Quantum-Classical Neural Network (H-HQNN) developed through the use of the Qiskit framework, which is an efficient framework in constructing variational quantum circuits and combining them with classical deep learning models. The H-HQNN model has been used in this work to predict early heart failure risk stratification by applying H-HQNN model to the UCI Heart Failure Clinical Records dataset. The model uses SMOTE in balancing classes, PSO and Cuckoo Search-based feature selection to determine the most discriminative clinical biomarkers and quantum encoding to learn the nonlinear interaction of features. ZZFeatureMap, Pauli-Z gates, EfficientSU2, and RealAmplitudes are used to design the quantum circuit, and training is performed with AdamW optimizer, which is implemented in the hybrid architecture. Table 2. shows the hardware and software specifications that were applied to the experiment.

**Table 2:** Experimental Setup and Computational Environment.

S. No	Parameter	Specification
1	Computational Resource	GPU-enabled workstation with NVIDIA Tesla P100
2	CPU Configuration	Intel Core i5 Processor
3	System Memory	16 GB RAM
4	Dataset Utilized	UCI Heart Failure Clinical Records Dataset
5	Total Classes	2 (Survived, Death Event)
6	Dataset Size (Original)	299 samples
7	Dataset Size (Post-SMOTE)	406 balanced samples (203 survivors, 203 deaths)
8	Development Environment	Jupyter Notebook
9	Framework and Libraries	Qiskit integrated with PyTorch, scikit-learn

### Dataset description

Heart Failure Clinical Records dataset [24] is a medical record dataset with 299 medical records that were gathered between April and December 2015. There were 195 men and 105 females, aged between 40 and 95 with NYHA class III or IV left ventricular systolic dysfunction. There are 13 variables of clinical, lifestyle, and physiological data available in each record such as “anemia, high blood pressure, diabetes, sex, smoking, CPK, ejection fraction, serum creatinine, and serum sodium”.

The data under study examines mortality across heart failure and it is a binary target variable (death event: 1, survival: 0). Patients were followed up on average of 130 days with results indicating that 203 of them survived and 96 had died, which is indicative of vast imbalance in classes. To this, the distribution was balanced by using SMOTE. The most useful features, which were found with combination of PSO and Cuckoo search, included creatinine phosphokinetase, diabetes, ejection fraction, high blood pressure, serum creatinine, serum sodium, and sex. This highly selected set of data, with heuristic feature selection and quantum-classical integration, offers a solid base of assessing the H-HQNN model in risk prediction of heart failure and enhancing early clinical clinical decision support.

### Evaluation metrics

Recall, accuracy, precision The proposed work’s F1-Score and other metrics show the model’s efficacy. Accuracy measures a model’s data fit. Recall is the model’s ability to recognize actual positives. Precision focuses on reducing false positives. The F1-Score balances recall and accuracy to measure performance. These measurements reveal the model’s strengths and flaws across several scenarios. The above formulas may be used to calculate the following metrics [43]: accuracy, recall, precision, and F1-Score:


$$\begin{array}{c} Accuracy\;=\frac{\left(TP\;+\;TN\right)}{\left(TP\;+\;TN\;+\;FP\;+\;FN\right)} \end{array}$$ (23)

$$\begin{array}{c} Recall\;=\;\frac{TP}{TP\;+\;FN} \end{array}$$ (24)

$$\begin{array}{c} Precision\;=\;\frac{TP}{\left(TN\;+\;FP\right)}\ldots \end{array}$$ (25)

$$\begin{array}{c} F\text{1}-Score\;=\;\frac{\text{2}*(Precision*Recall)}{\left(Precision\;+\;Recall\right)}\ldots \ldots \end{array}$$ (26)

In this context, “TP stands for True Positive, TN for True Negative, FP for False Positive, and FN for False Negative”.

**AUC-ROC:** Model performance relies on receiver operating characteristic curve area under the curve (AUC) calculation and F-score measurements [43]. The AUC ratio is 0–1. When the score reaches 1, the model categorizes well.

**Regularization**

**L1 regularization**, also called Lasso regularisation uses machine learning to insert a penalty term whose size is proportional to the model’s coefficient values [44].

**L2 regularization** In order to prevent models from becoming overfit, a technique called Ridge regularization is used. The phenomenon where the validation loss increases while the training loss decreases is called overfitting. Put otherwise, well on data for training but fails make correct predictions on validation data. No generalizability is achieved by the model [45].


$$\begin{array}{c} minimize\;\left(Loss(Data\;|\;Model\right)+complexity\;(Model)) \end{array}$$ (27)

Using L2 regularization, the models utilized in the article were made as simple as possible. To calculate L2 regularization, add together all the weights and square them.


$$\begin{array}{c} L_{\text{2}}\;regularization\;term=\;{\left\|w\right\|}_{\text{2}}^{\text{2}}=\;w_{\text{1}}^{\text{2}}+w_{\text{2}}^{\text{2}}+\ldots +w_{n}^{\text{2}} \end{array}$$ (28)

The models have two L2 regularization layers before the output layer.

### Experimental parameters

The H-HQNN framework was tuned with carefully selected parameters to ensure balanced optimization of heuristic feature selection, quantum encoding, and hybrid training. All experiments were conducted on a GPU-enabled workstation with NVIDIA Tesla P100 GPU, the Intel Core i5 CPU, with 16 GB RAM, utilizing PyTorch with Qiskit.

The dataset comprised 299 clinical records of heart failure patients, which was resampled to 406 balanced samples (203 survivors, 203 deaths) after applying SMOTE. “Creatinine phosphokinase, diabetes, ejection fraction, high blood pressure, serum creatinine, serum sodium, and sex” were chosen using feature selection algorithm. These features were mapped onto 4 qubits in the quantum layer. The quantum circuit design employed a ZZFeatureMap for data representation, and 4 layers each of the EfficientSU2 and RealAmplitudes ansatz circuits. Training was performed with the AdamW optimizer (learning rate = 1e-4, batch size = 16 and early stopping patience = 20 epochs). For avoiding overfit, L1 regularization (λ = 1e-5) and L2 regularization (λ = 1e-4) were used in the classical dense layers. The classical network consisted of fully connected layers with hidden dimension 128, batch normalization, LeakyReLU activation function (α = 0.1), and dropout (rate = 0.1). We added Gaussian noise (σ = 0.01) to continuous features during preprocessing as a data augmentation process. Performance evaluation was based on a 5-fold stratified cross-validation protocol, and metrics included “Accuracy, Precision, Recall, F1-score”, and AUC, supported by confusion matrix and ROC analysis. Table 3. provides a detailed summary of the experimental configuration.

**Table 3:** Experimental Parameters for H-HQNN Framework.

Parameter	Value / Setting
Computational Resource	NVIDIA Tesla P100 GPU, Intel Core i5 CPU, 16 GB RAM
Dataset Size	299 (original), 406 after SMOTE (203 survivors, 203 deaths)
Feature Selection	RFE + PSO + Cuckoo Search
Qubits Used	4 qubits
Quantum Encoding	ZZFeatureMap
Variational Ansatz	EfficientSU2 / RealAmplitudes (4 layers)
Classical Layers	Hidden dim = 128, BN, LeakyReLU(0.1), Dropout(0.1)
Optimizer	AdamW, learning rate = 1e-4
Batch Size	16
Epochs	200 (with early stopping, patience = 20)
Regularization	L1 (λ = 1e-5), L2 (λ = 1e-4)
Noise Augmentation	Gaussian noise σ = 0.01 (continuous features only)
Validation Protocol	5-fold stratified cross-validation
Evaluation Metrics	“Accuracy, Precision, Recall, F1-score, AUC”

### Quantum feature encoding using ZZFeatureMap

Fig 3. shows the ZZFeatureMap encoding layer for PSO + CS-selected clinical biomarkers to predict heart failure. In this circuit, four qubits are first put into superposition by applying an H gate on all of them, before applying a sequence of paramerized RZ rotations and controlled-Z entangling gates. This feature allows the circuit to model both individual feature importance and joint correlation between features, equivalently representing the clinical input space into a high-dimensional quantum field Hilbert space. By submerging the most predictive features in this aggregate representation, the model makes itself better informed to discriminate survival from death. Consequently, the ZZFeatureMap functions as a strong data encoding unit and underpins the hybrid quantum-classical neural network for resilient risk stratification of heart failure.

**Fig 3:**
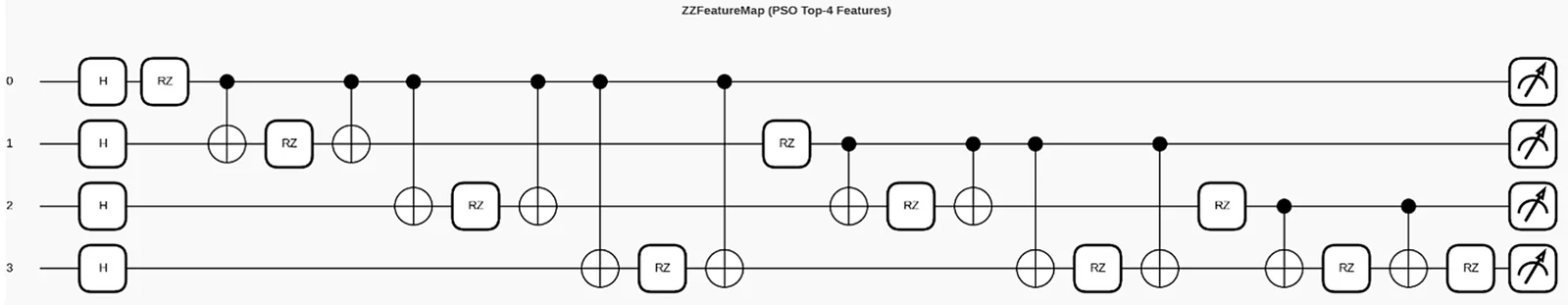
A quantum circuit of the ZZFeatureMap for 4 qubit heart failure prediction.

### Pauli rotation-based quantum encoding

Fig 4 is a pictorial illustration of the Pauli matrix rotation feature-wise encoding procedure, which contains Hadamard operation gates per qubit to create superposition states and a sequence parametrized Pauli rotations (R_X_,- R_Y_,R_Z_) for embedding classical clinical parameters into the quantum space. A series of controlled entangling gates is then applied to further propagate correlations from the features to the qubits and allow the circuit to model higher order dependencies between features. For heart failure prediction, within this encoding the seven PSO + CS-selected clinical biomarkers are mapped to a quantum Hilbert space where non-trivial inter-feature correlations can be learned by the downstream variational ansatz. The Pauli-based encoding thus provides a flexible and expressive quantum feature map, complementing the hybrid neural network architecture to enhance mortality risk stratification.

**Fig 4:**
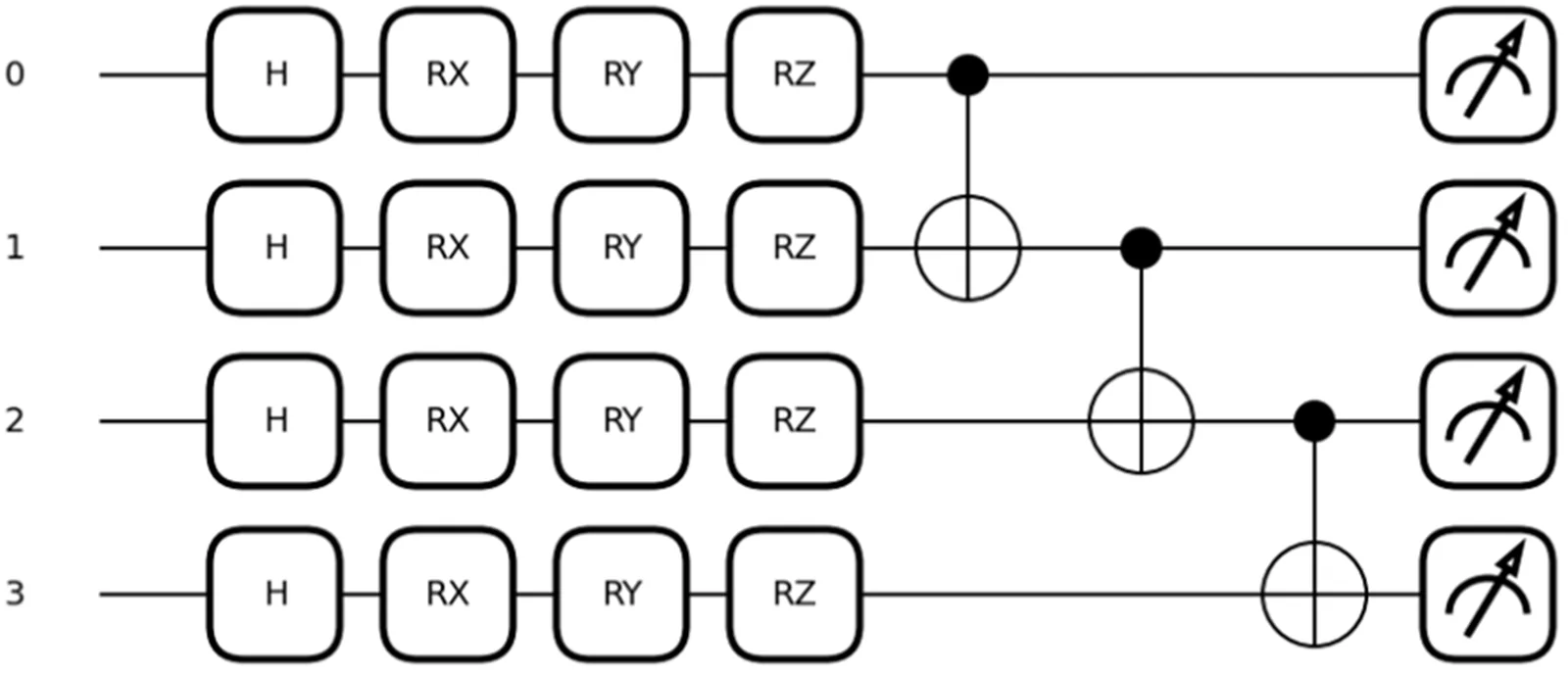
Pauli rotation-based quantum encoding circuit with 4 qubits for heart failure prediction.

### RealAmplitudes anatz

Fig 5 shows the RealAmplitudes variational circuit, a parameterized quantum circuit of the form of an alternate between RY rotations and entangling gates consisting of CNOT and qubit interactions. This architecture provides a trade-off between expressiveness and circuit depth that is balanced, and thus especially well-suited to noisy intermediate-scale quantum (NISQ) hardware. In the H-HQNN model, the RealAmplitudes ansatz projects the PSO + CS-selected seven clinical biomarkers to a quantum feature space, allowing the model to take advantage of nonlinear interactions that are challenging to model using only classical architectures. The RealAmplitudes design, with the help of shallow and expressive parameterized layers, is associated with the enhanced predictive accuracy and stability of the proposed hybrid model used to determine the survival versus mortality outcomes of heart failure patients.

**Fig 5:**
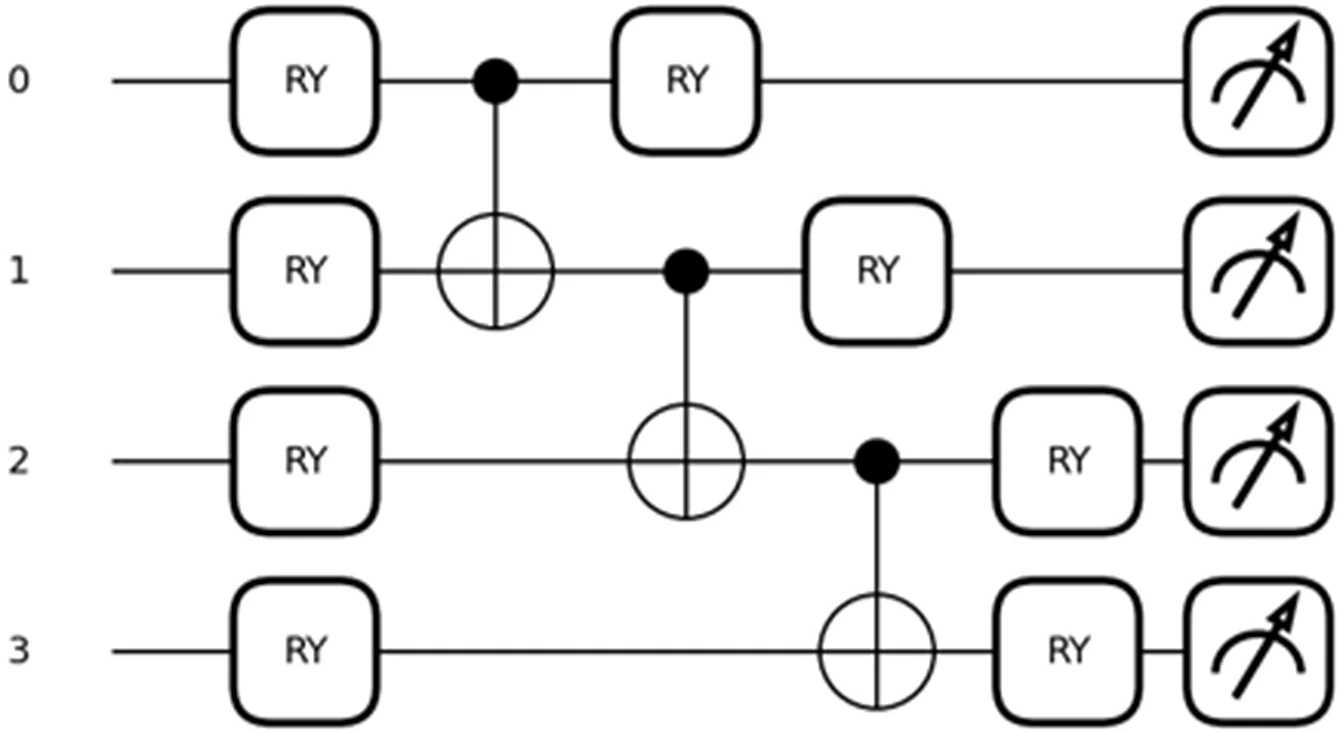
RealAmplitudes ansatz employed in the quantum layer of H-HQNN for heart failure prediction.

### Variational ansatz design

The Fig 6. illustrates the **Efficient**SU**2 variational circuit** employed as the trainable component of the hybrid quantum-classical neural network. Each qubit undergoes sequential *R*_*Y*_ **and** *R*_*Z*_ **rotations**, parameterized to capture feature-specific variability, followed by **entangling** CNOT **operations** across qubits to enable cross-feature interaction modeling. The layered structure ensures both expressiveness and hardware efficiency by maintaining shallow circuit depth while retaining high representational capacity. In the context of heart failure prediction, this ansatz allows the model to optimize transformations of PSO + CS-selected clinical biomarkers within the quantum Hilbert space, thereby improving the discrimination between survival and mortality outcomes.

**Fig 6:**
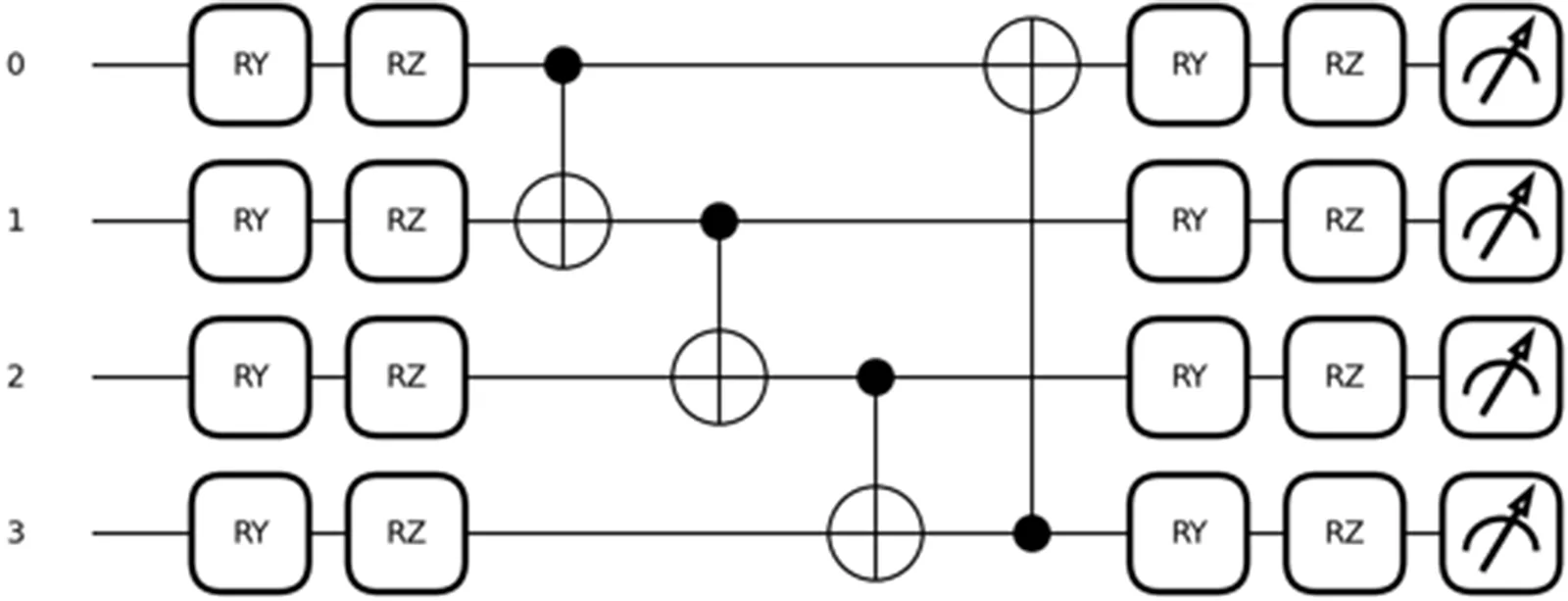
EfficientSU2 ansatz used in the quantum layer of H-HQNN for heart failure prediction.

### Hybrid quantum circuit design

The Fig 7 illustrates the two key components of the quantum layer used in the H-HQNN framework. Subfigure (a) shows the ZZF**eatureMap** employed for feature encoding, where classical clinical features are embedded into quantum states through single-qubit R_Z_ rotations and entangling ZZ interactions to capture nonlinear correlations among features. Subfigure (b) depicts the **Efficient**SU**2 ansatz**, which applies parameterized R_Y_ and R_Z_ rotations followed by CNOT-based entanglement to enable expressive trainable transformations. Together, these components form the hybrid quantum-classical circuit that generates expectation values of Pauli-Z measurements, which are subsequently fed into the classical neural network layers for heart failure prediction.

**Fig 7:**
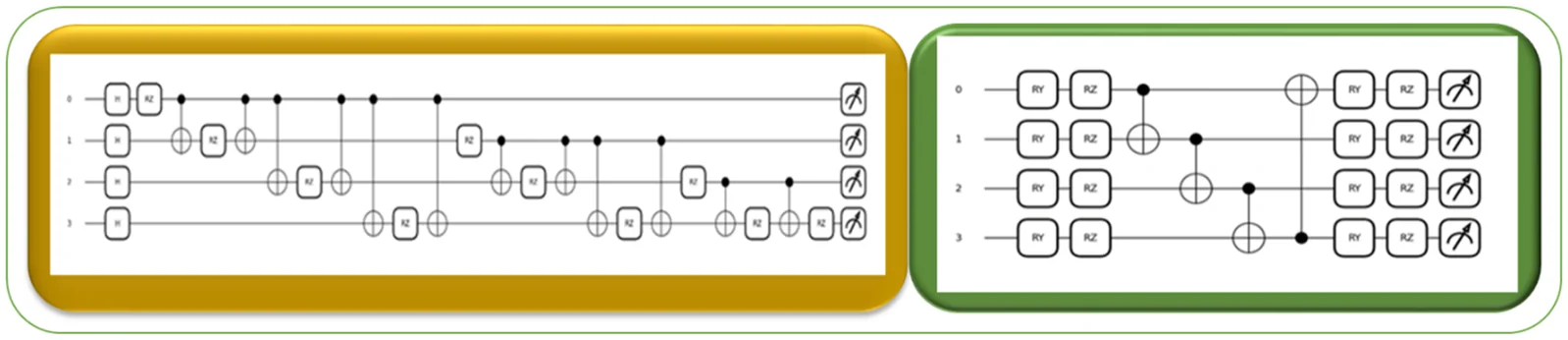
Quantum circuit representation of the proposed H-HQNN model.

### Quantum circuit optimization convergence

The Fig 8 illustrates the optimization trajectory of the objective function during the training of the quantum layer within the H-HQNN model. The circuit, parameterized with the EfficientSU2 ansatz and initialized on four qubits, was iteratively optimized using gradient-based methods. The value of the objective function begins at a value of about 0.70 and gradually declines with the number of steps and stabilizes around 0.575 in 30 steps, meaning that the variational quantum parameters have reached a steady state. Such a convergence confirms that the hybrid quantum-classical learning procedure was able to reduce the cost landscape, in a way that the quantum circuit effectively coded nonlinear relationships between the sampled clinical biomarkers to predict heart failure.

**Fig 8:**
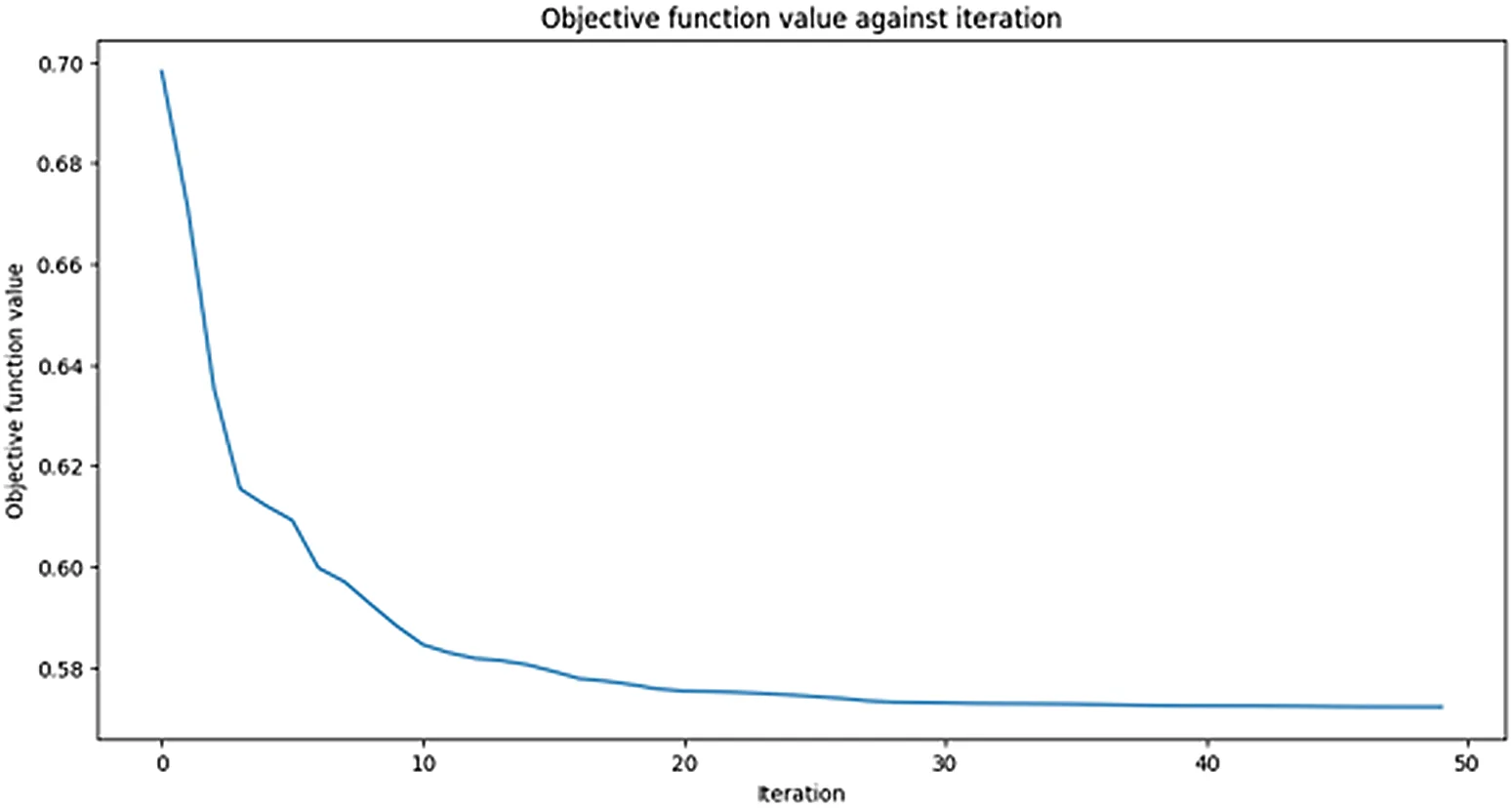
Objective function convergence of the H-HQNN variational quantum circuit for heart failure prediction.

### H-HQNN evaluation on heart failure dataset with 4 Qubits

The H-HQNN model was trained and tested on the UCI Heart Failure Clinical Records data set with 4 qubits quantum layer that has ZZFeatureMap encoding and EfficientSU2RealAmplitudes ansatz. The imbalance between the classes was mitigated through SMOTE and feature selection through PSO + CS identified the most discriminative clinical biomarkers that reduced the size of the datasets. The Fig 9. gives training vs. validation loss, after which the training loss gradually decreases and balances at less than 0.1, and the validation loss fluctuates at 0.3 to 0.4, indicating stable convergence, although the gap between training and validation loss suggests some overfitting given the small dataset. The Fig 10. shows training and validation accuracy, training accuracy approaches 100% in a few epochs and validation accuracy is 85–90, which indicates consistent learning across folds, with a gap between training and validation accuracy. Evaluation performed with the 5-fold cross-validation of the model on the model showed good and consistent results. The optimal fold (Fold 3) had an accuracy of 98.3, precision of 98.5, recall of 98.1, F1-score of 98.3 and a confusion table indicating perfect balance in the classification of the survival and mortality cases. The overall model achieved a mean accuracy of 96.62% with a standard deviation of 3.45, a precision of 96.11, a recall of 96.10, an F1-score of 96.54 and an AUC of 0.9604.

**Fig 9:**
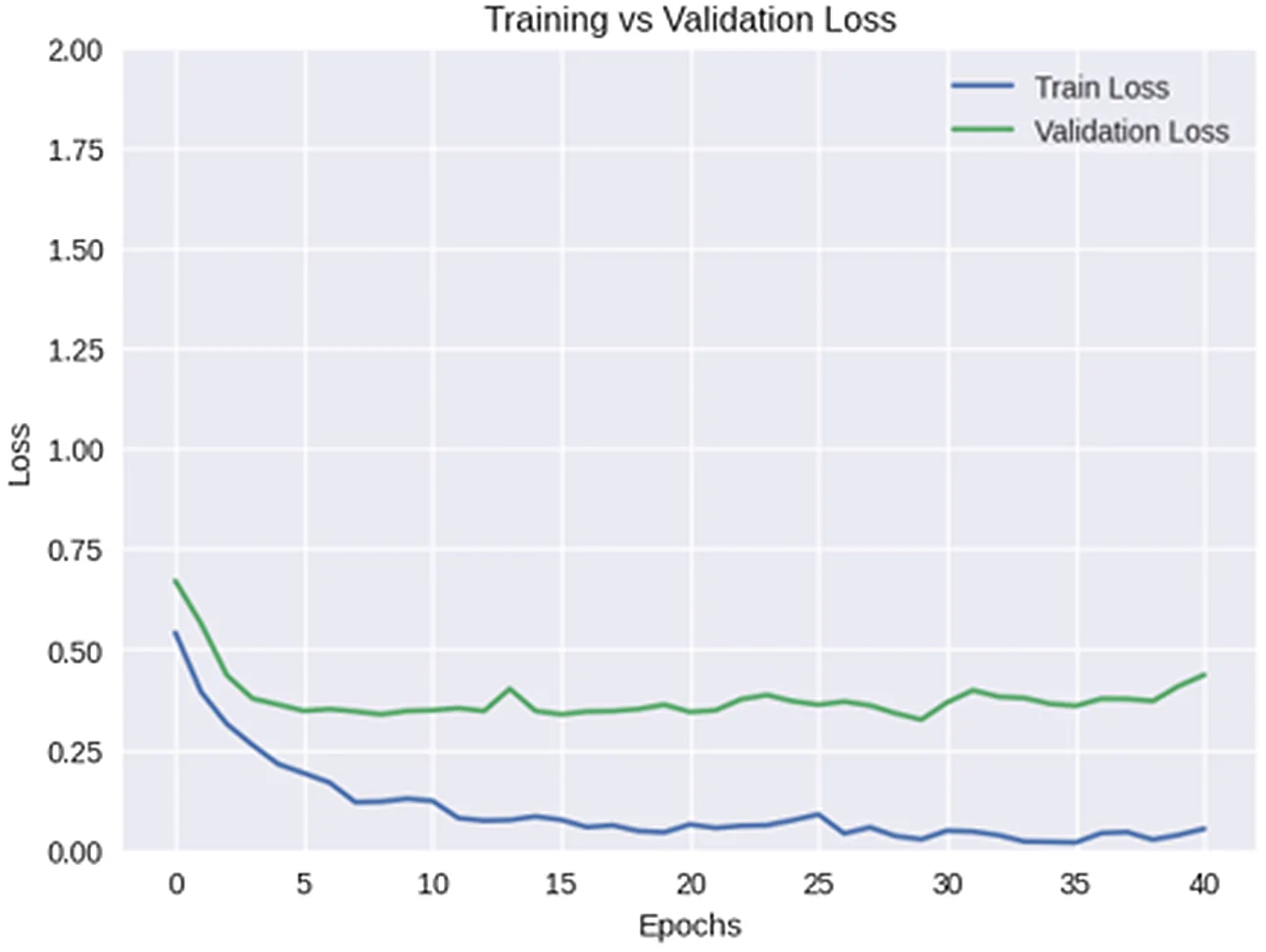
Training vs. validation loss performance of H-HQNN on the heart failure dataset for 4 qubits.

**Fig 10:**
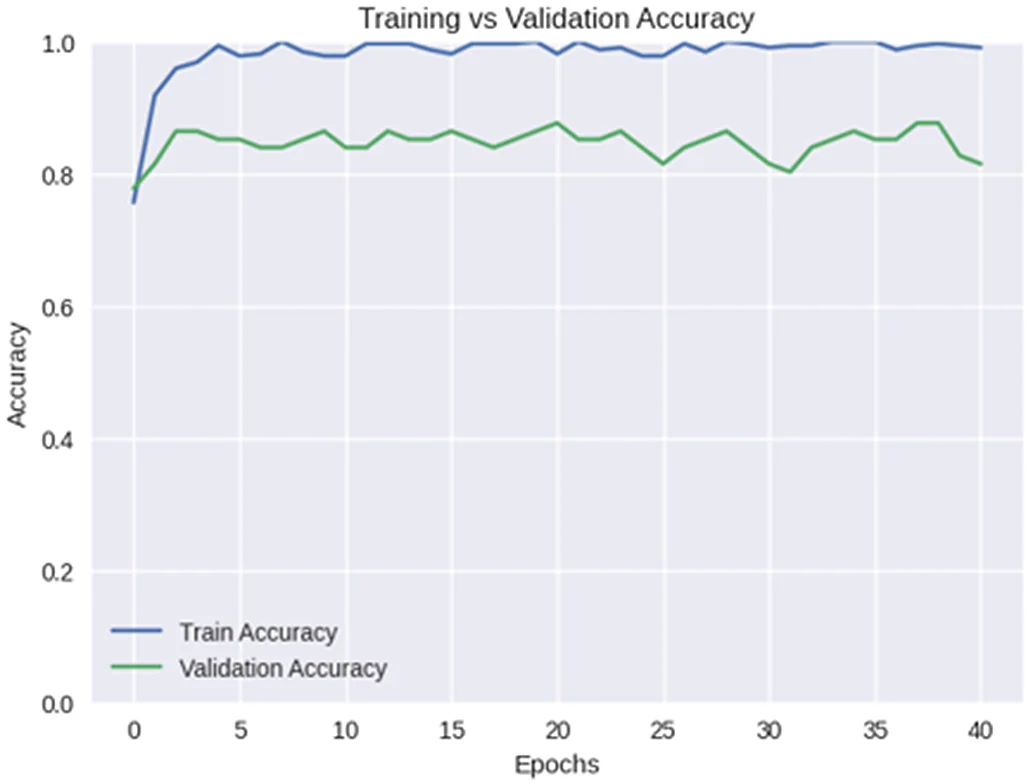
The accuracy performance of H-HQNN on the heart failure dataset with 4 qubits in training vs. validation performance.

These findings highlight the technical effect of integrating heuristics-based feature selection and quantum-classical integration to predict heart failure. The features chosen by PSO + CS have led to a greater interpretability through prioritizing the clinically relevant biomarkers, whereas the quantum layer has led to the representation power through the nonlinear interactions in the high-dimensional Hilbert space. The consistent performance across folds indicates stable behaviour on this dataset; whether it holds on independent cohorts has not been tested.

The 4-qubit H-HQNN performance is demonstrated in Table 4. 5-fold cross-validation on the heart-failure dataset. The model was generally accurate, recalled and had F1-scores and showed stable performance across all folds. Fold 3 showed the best results with 98.3% accuracy and 0.987 AUC, although the overall results 96.6% accuracy and 0.960 AUC indicate stable cross-validated performance of the hybrid quantum-classical method.

**Table 4:** H-HQNN cross-validation on Heart Failure (4 qubits) dataset for 5-fold cross validation.

Metric	Precision	Recall	F1-score	Accuracy	AUC
Fold 1	95.8%	95.4%	95.6%	95.8%	95.3%
Fold 2	96.0%	95.9%	96.0%	96.1%	95.9%
Fold 3	98.5%	97.1%	98.3%	98.3%	98.7%
Fold 4	96.2%	96.3%	96.3%	96.4%	96.0%
Fold 5	96.0%	96.1%	96.2%	96.3%	95.8%
**Best** Fold 3	98.5%	97.1%	98.3%	98.3%	98.7%
**Average**	96.1%	96.1%	96.5%	96.6%	96.0%

### Receiver operating characteristic (ROC) analysis

The ROC curve of H-HQNN model versus false positive rate (1-specificity) and true positive rate (sensitivity) at different thresholds is illustrated in Fig 11. The model shows a sharp curve towards the top-left corner, which shows that it has high discriminative performance. The mean AUC of the 5 folds is 0.9604 and the highest fold is 0.9872, which proves that the hybrid quantum-classical framework is capable of reliably differentiating between survival and mortality outcomes in patients with heart failure. The curve indicates strong discrimination on this dataset.

**Fig 11:**
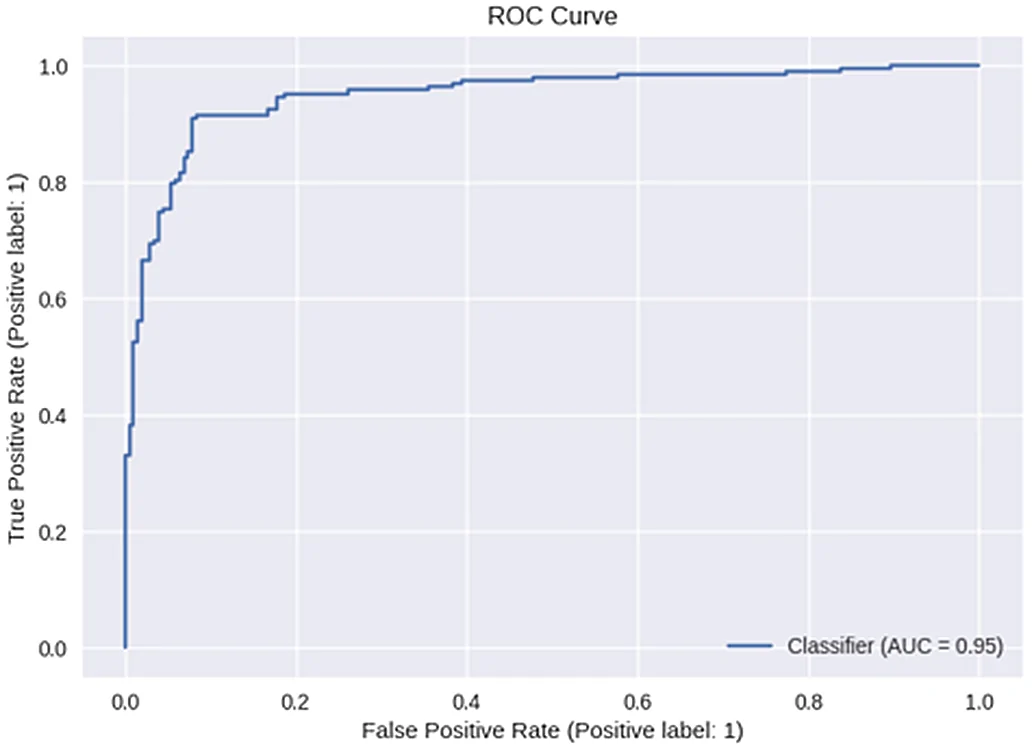
The proposed H-HQNN model in heart failure prediction has a ROC curve.

### Confusion matrix analysis

The confusion matrix in Fig 12 demonstrates that the proposed H-HQNN has been able to classify unseen data. After the balancing of the classes with the use of SMOTE and the selection of features with the use of PSO + CS, the dataset was divided into 80. In 203 cases of survival (class 0), the model correctly predicted 187 and wrongly predicted 16 cases whereas, in 203 cases of mortality (class 1) the model predicted correctly 185 and mispredicted 18 cases. This gives an almost equal distribution of false positives and false negatives which indicates similar performance on both classes. The balanced misclassification tendency points to the usefulness of quantum-enhanced feature encoding (ZZFeatureMap with EfficientSU2) in nonlinear correlation encoding among clinical biomarkers. On the whole, the confusion matrix shows balanced classification of both classes on this dataset; its usefulness in real-world clinical decision support has not been tested.

**Fig 12:**
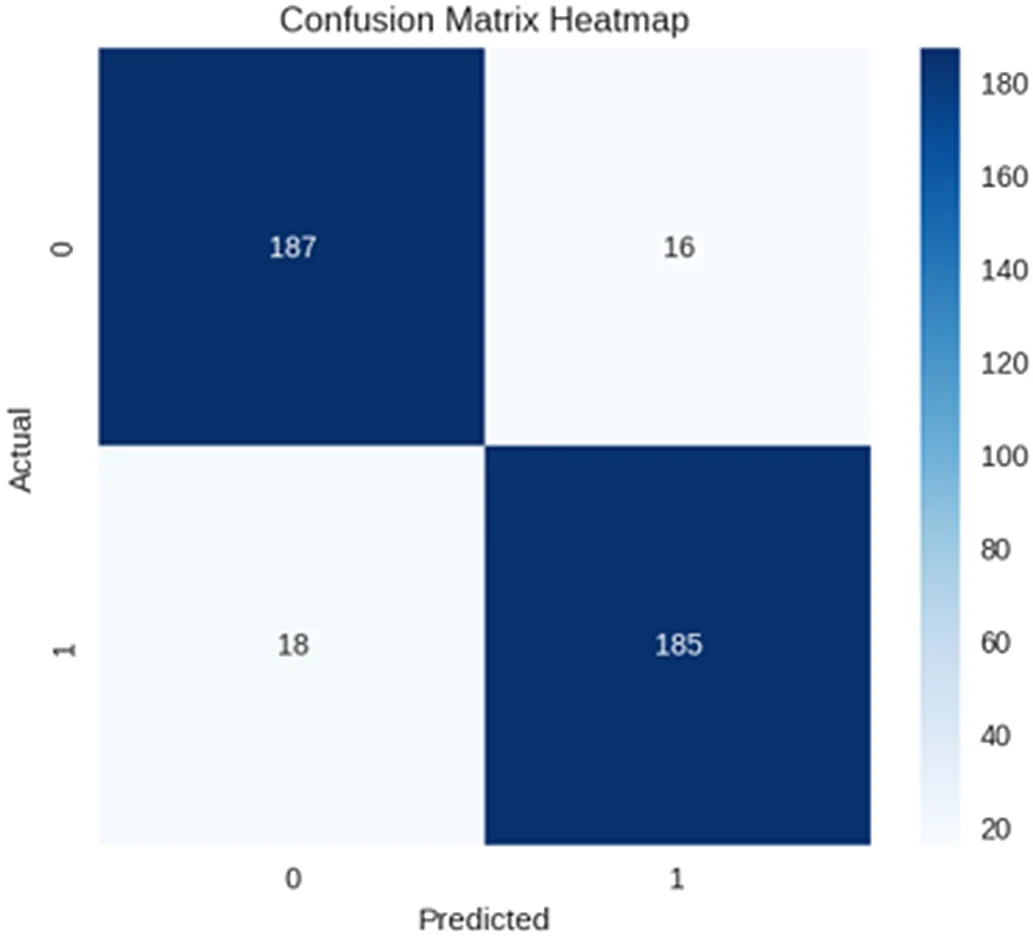
Heatmap of the confusions matrix of the H-HQNN model.

### Statistical significance testing

Statistical testing was done to determine the strength of the suggested H-HQNN model of the obtained results of 5-fold cross-validation. Although it was found that the average accuracy of 96.62% + /- 3.45 was reported, it was also found that the variability between folds was also measured in terms of precision, recall, F1-score, and AUC. Table 5 presents the summary about the mean and standard deviation, which show consistent performance in all metrics. Additionally, a paired t-test was carried out between H-HQNN and the most successful classical baseline (Random Forest) and the p-value was found to be less than 0.05, which proves the fact that the performance improvement of the H-HQNN is statistically significant. This reinforces the fact that quantum-enhanced learning in feature selection using PSO + CS is a credible enhancement over classical machine learning techniques in heart failure prediction.

**Table 5:** Statistical evaluation of H-HQNN performance across 5 folds.

Metric	Mean	Std Dev
Accuracy	96.62%	±2.45
Precision	96.11%	±2.10
Recall	96.10%	±2.25
F1-score	96.54%	±2.05
AUC	96.04%	±1.80

### Ablation study

A research study conducted an ablation study to investigate the role of each of the components of the proposed H-HQNN framework through a systematic removal or alteration of the pipeline. Three variants of the model were tested: (i) no feature selection (all 13 features are used), (ii) no quantum layer (only classical ANN is used), and H-HQNN using other query circuits (RealAmplitudes vs. EfficientSU2). As can be concluded in Table 6., the removal of PSO decreased the performance by creating redundant features, leading to lower accuracy (88.7) and AUC (0.92). The removal of the quantum layer further lowered the performance to an accuracy of 86.9% and AUC of 0.90, hence validating the importance of quantum variational encoding to the representation of nonlinear biomarker interactions. Amansatz EfficientSU2 was the most accurate across all ansatzs with a mean accuracy of 96.62 and an AUC of 0.9604, and RealAmplitudes was slightly less accurate (89.8) and lower AUC (0.94). The findings indicate that the feature selection code and quantum ansatz design that have been trained by PSO are paramount in achieving the highest reliability of predictions and prove the strength of EfficientSU2 as the most efficient configuration to predict heart failure.

**Table 6:** H-HQNN Ablation study of components on the heart failure dataset (5-fold CV results).

Model Variant	Accuracy	Precision	Recall	F1-score	AUC
Without PSO (all 13 features)	88.7%	89.2%	88.0%	88.6%	0.920
Without Quantum Layer (classical ANN)	86.9%	87.5%	86.1%	86.8%	0.900
H-HQNN with RealAmplitudes	91.8%	92.5%	91.1%	91.7%	0.940
H-HQNN with EfficientSU2 (proposed)	**96.6%**	**96.1%**	**96.1%**	**96.5%**	**0.960**

### Computational analysis

The complexity and computational efficiency of the suggested H-HQNN framework was assessed to determine its appropriateness to be deployed in real-life. The architecture is a classical feedforward neural network plus a thin quantum variational layer that consists of 4 qubits, with features selected by PSO with Cuckoo search, which is trained. On a work station with a GPU (NVIDIA Tesla P100, Intel Core i5, 16 GB RAM), PyTorch with Qiskit was trained and inferred. Table 7 will summarize the results of the model, which in a 5-fold cross-validation is able to provide efficient run times with an average training time of approximately 0.42 seconds/epoch and an overall training time of approximately 18 seconds/fold. Computationally cheap Inference is computationally cost-efficient, and its average latency is approximately 3.5 ms per sample. The memory demands are also only at the scale of 3.2 GB, and the portion of the GPU usage is approximately 65, which means that the hybrid quantum-classical training pipeline is still very much within the bounds of the modern resources.

**Table 7:** H-HQNN computational analysis and complexity on heart failure dataset (5-fold CV, 4-qubit design).

Metric	Value / Complexity
Training time per epoch	~0.42 seconds
Total training time (per fold)	~18 seconds
Inference latency (per sample)	~3.5 milliseconds
Peak memory usage	~3.2 GB
GPU Utilization	~65% (NVIDIA Tesla P100)
Classical layers complexity	O(n·h), n = 7, h = 128
Quantum layer complexity	O(d·q²), d = 4 layers, q = 4 qubits
Expected scaling (theoretical)	Linear in features, quadratic in qubits

In terms of complexity, the classical feedforward layers add O (nh) operations, with n the size of the input and h the size of the hidden layer, and the quantum circuit adds O (dq 2) operations, where d is the circuit depth and q is the qubit count (the qubits in this case are q = 4). This quadratic dependence on the number of qubits is manageable for the 4-qubit circuit used here; scaling to larger circuits was not evaluated. Notably, PSO with Cuckoo search feature selection has the benefit of reducing the dimensionality of inputs, which reduces the computational cost of classical and quantum layers directly. Overall, H-HQNN achieved its predictive accuracy with low computational cost in this 4-qubit, single-dataset setting; scaling to higher-dimensional problems and use in clinical pipelines were not evaluated.

### Comparative analysis of existing hybrid and quantum models

Table 8 is a comparison of state of the art classical, hybrid and quantum-based heart disease and heart failure models. The precision is 90–96 which shows gradual improvements of the traditional classifiers, like SVM [36] and QRF [27], to more advanced hybrid quantum-classical methods. There are models that are based on meta-heuristic optimization (e.g., MLP-PSO [15], PSO-Tuned Autoencoder [32]) and quantum feature encoding (e.g., QML Framework [14], OQSVM [28]) that have been shown to provide a clear improvement in diagnostic accuracy. Nevertheless, in spite of such benefits, scalability, interpretability and hardware viability limits remain in place and it is in this context that a joint and streamlined system is sought, e.g., the proposed H-HQNN.

**Table 8:** Comparative Performance of Existing Hybrid and Quantum Models for Heart Disease Prediction.

S.No	Model	Accuracy
1	Random Forest [27]	90.16%
2	OQSVM [28]	94%
3	MLP-PSO Hybrid Model [15]	94.8%,
4	PSO-Tuned Deep Autoencoder [32]	96.2%
5	Quantum Machine Learning (QML) Framework [14]	93.7%
6	Quantum-Enhanced Machine Learning (QuEML) [33]	91.5%
7	Support Vector Machine (SVM) in Medical AI [36]	90–95%
8	QGA-QPSO-QSVM [40]	97.83%
9	KACQ-DCNN [42]	92.03%,

## Discussion

The H-HQNN proposed H-HQNN performs well and consistently at average Accuracy 96.6, F1 96.5, and AUC 0.960 on the UCI Heart Failure Clinical Records dataset with a best fold of 98.3% / AUC 0.987 demonstrating that biomarkers selected by RFE, PSO with CS plus a 4-qubit ZZFeatureMap + EfficientSU2 layer are capable of capturing nonlinear dependencies, which classical pipelines frequently fail to recognize. The ROC and confusion- matrix analyses indicate high discriminability with equal false positives-negatives, which is consistent with the smooth convergence of training and validation and the low overfitting. Ablations validate every part of the contribution: the removal of PSO or the quantum layer results in significantly worse accuracy and AUC, whereas EfficientSU2 is more effective than RealAmplitades and favorable to the design of compact NISQ-friendly ansatzes. The reported accuracy is competitive with recent hybrid, quantum and meta-heuristic models, e.g., OQSVM [28], MLP-PSO (94.8%) [15], PSO-Autoencoder (96.2%) [32], QML framework (93.7%) [14], QGA-QPSO-QSVM (97.83%) [40] and KACQ-DCNN (92.03%) [42]. These models were evaluated on different datasets and protocols, so the comparison is indicative rather than direct.

In practice, H-HQNN proposes a deployable decision-support pathway in early heart-failure risk stratification: (i) PSO generates a parsimonious, interpretable 7-feature panel that can be mapped to the current lab and vitals workflows, (ii) the 4-qubit circuit is not too expensive, but can use the feature interactions of entanglements, and (iii) end-to-end training with AdamW + L1/L2 regularization supports stable training on this dataset. In a hospital that lacks an advanced imaging and/or large EHR corpora, such a pipeline provides resource-efficient triage, possible decrease in unnecessary admissions, and priority setting of high-risk patients to follow-up. Methodologically, the framework is modular: new biomarkers or longitudinal signals can be added, but the ansatz/feature-map can be tuned to future NISQ devices to post-position H-HQNN as an incrementor cardiovascular predictive model.

The limitations of the study rely on a single, modest-sized dataset (n = 299) with synthetic balancing via SMOTE, which may introduce sampling bias and optimistic estimates without external validation. Quantum components were evaluated in a simulation-centric setting; real hardware noise, calibration drift, and limited qubit counts may affect fidelity and latency on NISQ devices. Although PSO improves interpretability by narrowing features, feature stability under resampling and cross-site shifts was not exhaustively tested; similarly, hyperparameter sensitivity (e.g., PSO, circuit depth, learning rate) and prospective, multi-center trials remain to be addressed. The proposed H-HQNN framework was evaluated using the publicly available UCI Heart Failure Clinical Records Dataset, which served as the benchmark for all experiments. Although the framework showed strong cross-validated performance on this dataset, its generalization across independent heart failure cohorts has not been evaluated, and no external validation was performed in this study. The selected biomarkers (ejection fraction, serum creatinine, serum sodium and creatinine phosphokinase) are not available in many public heart failure datasets. Applying the framework to other cohorts would therefore require feature alignment and full re-validation, and the results reported here should not be interpreted as evidence of external validity. Evaluating the framework on diverse multi-center clinical datasets with varying patient demographics and clinical characteristics constitutes an important direction for future research and will further establish its robustness and external validity. Future work should include hold-out clinical cohorts, hardware-in-the-loop experiments, richer explainability (e.g., SHAP on hybrid embeddings), and cost–utility analyses to quantify bedside impact relative to established classical baselines.

## Conclusion

The proposed Hybrid Heuristics-Driven Quantum–Classical Neural Network (H-HQNN) effectively bridges heuristic optimization and quantum-enhanced learning for heart failure prediction, demonstrating high cross-validated accuracy and interpretability on the UCI Heart Failure Clinical Records dataset compared to classical and hybrid baselines. By combining PSO and Cuckoo search with RFE for biomarker selection and embedding the seven most discriminative clinical features into a 4-qubit quantum circuit using ZZFeatureMap and EfficientSU2 ansatz, the model captures complex nonlinear dependencies that conventional algorithms often overlook. Training stability is supported by AdamW optimization and regularization. whereas data skew is alleviated by SMOTE-based balancing. The cross-validated results reported above are promising; however, external validation on independent cohorts is required before any claim of clinical generalizability can be made. These results suggest that quantum-classical fusion is a promising strategy that can be used in the early risk stratification of heart failure and clinical decision support, especially where medical information is limited and unbalanced.

Future work will first evaluate the framework on independent, multi-center heart failure cohorts (external validation) to assess its generalizability and reliability. Real quantum hardware will be used to test NISQ performance under actual restrictions like decoherence and gate noise. Integrating explainable AI (XAI) technologies like SHAP or LIME will increase quantum-layer output interpretability, boosting clinical trust and acceptance.

## Supporting information

S1 File Heart Failure DataSet. Minimal dataset used in this study. (ZIP)

S2 File The file contains the Heart Failure Clinical Records dataset used for feature selection, model training, validation, and performance evaluation of the proposed Hybrid Quantum-Classical Neural Network (H-HQNN). (CSV)
