# Supplementary material for: H-HQNN: Hybrid quantum-classical neural network with feature selection by heuristics to predict heart failure
Source: PLoS One. 2026 Oct 16;21(10):e0360391. doi: 10.1371/journal.pone.0360391 (PMC13674744; doi:10.1371/journal.pone.0360391)
Supplement: Supplementary file 1 [file pone.0360391.s001.zip › Heart Failure DataSet/Heart Failure data Set/Heart_Failure_Clinical_Records_dataset description.docx]

# Heart Failure Clinical Records Data Dictionary

**Title:** Data Dictionary for the **Heart Failure Clinical Records**

**Donated on 2/4/2020**

This dataset contains the medical records of 299 patients who had heart failure, collected during their follow-up period, where each patient profile has 13 clinical features.

**Dataset Characteristics**

Multivariate

**Subject Area**

Health and Medicine

**Associated Tasks**

Classification, Regression, Clustering

**Feature Type**

Integer, Real

**# Instances**

299

**# Features**

12

**Dataset Information**

**Additional Information**

A detailed description of the dataset can be found in the Dataset section of the following paper: Davide Chicco, Giuseppe Jurman: "Machine learning can predict survival of patients with heart failure from serum creatinine and ejection fraction alone". BMC Medical Informatics and Decision Making 20, 16 (2020). https://doi.org/10.1186/s12911-020-1023-5

**Has Missing Values?**

No

**Introductory Paper**

[**Machine learning can predict survival of patients with heart failure from serum creatinine and ejection fraction alone**](https://www.semanticscholar.org/paper/e64579d8593140396b518682bb3a47ba246684eb)

By D. Chicco, Giuseppe Jurman. 2020

Published in BMC Medical Informatics and Decision Making

**Variables Table**

| **Variable Name** | **Role** | **Type** | **Demographic** | **Description** | **Units** | **Missing Values** |
| --- | --- | --- | --- | --- | --- | --- |
| age | Feature | Integer | Age | age of the patient | years | no |
| anaemia | Feature | Binary |  | decrease of red blood cells or hemoglobin |  | no |
| creatinine_phosphokinase | Feature | Integer |  | level of the CPK enzyme in the blood | mcg/L | no |
| diabetes | Feature | Binary |  | if the patient has diabetes |  | no |
| ejection_fraction | Feature | Integer |  | percentage of blood leaving the heart at each contraction | % | no |
| high_blood_pressure | Feature | Binary |  | if the patient has hypertension |  | no |
| platelets | Feature | Continuous |  | platelets in the blood | kiloplatelets/mL | no |
| serum_creatinine | Feature | Continuous |  | level of serum creatinine in the blood | mg/dL | no |
| serum_sodium | Feature | Integer |  | level of serum sodium in the blood | mEq/L | no |
| sex | Feature | Binary | Sex | woman or man |  | no |

**Additional Variable Information**

Thirteen (13) clinical features: -

age: age of the patient (years)

anaemia: decrease of red blood cells or hemoglobin (boolean)

- creatinine phosphokinase (CPK): level of the CPK enzyme in the blood (mcg/L)

- diabetes: if the patient has diabetes (boolean)

- ejection fraction: percentage of blood leaving the heart at each contraction (percentage) - high blood pressure: if the patient has hypertension (boolean)

- platelets: platelets in the blood (kiloplatelets/mL)

- sex: woman or man (binary)

- serum creatinine: level of serum creatinine in the blood (mg/dL) - serum sodium: level of serum sodium in the blood (mEq/L)

- smoking: if the patient smokes or not (boolean)

- time: follow-up period (days) - [target] death event: if the patient died during the follow-up period (boolean)

For more information, please check Table 1, Table 2, and Table 3 of the following paper: Davide Chicco, Giuseppe Jurman: "Machine learning can predict survival of patients with heart failure from serum creatinine and ejection fraction alone". BMC Medical Informatics and Decision Making 20, 16 (2020). https://doi.org/10.1186/s12911-020-1023-5

## Dataset Description

The dataset comprises clinical records of 299 patients diagnosed with heart failure who were followed over time to evaluate survival outcomes. It contains 13 variables, including demographic characteristics, comorbidities, laboratory measurements, clinical parameters, and one outcome variable. The dataset contains no missing values. The outcome variable, DEATH_EVENT, indicates whether the patient died during the follow-up period.

| **Variable Name** | **Data Type** | **Description** | **Units/Coding** |
| --- | --- | --- | --- |
| age | Integer | Age of the patient at baseline. | Years |
| anaemia | Binary | Indicates whether the patient had anaemia (decreased red blood cell count or hemoglobin level). | 0 = No, 1 = Yes |
| creatinine_phosphokinase | Integer | Serum creatinine phosphokinase (CPK) enzyme concentration. | mcg/L |
| diabetes | Binary | Indicates whether the patient had diabetes mellitus. | 0 = No, 1 = Yes |
| ejection_fraction | Integer | Percentage of blood ejected from the left ventricle during each cardiac contraction. | % |
| high_blood_pressure | Binary | Indicates whether the patient had hypertension. | 0 = No, 1 = Yes |
| platelets | Continuous | Platelet count measured in peripheral blood. | kiloplatelets/mL |
| serum_creatinine | Continuous | Serum creatinine concentration, a marker of renal function. | mg/dL |
| serum_sodium | Integer | Serum sodium concentration. | mEq/L |
| sex | Binary | Biological sex of the patient. | 0 = Female, 1 = Male |
| smoking | Binary | Indicates whether the patient was a smoker. | 0 = No, 1 = Yes |
| time | Integer | Duration of follow-up from baseline until death or censoring. | Days |
| DEATH_EVENT | Binary (Outcome) | Patient survival status during the follow-up period. | 0 = Survived, 1 = Died |

## Dataset Summary

- Dataset Name: Heart Failure Clinical Records
- Number of Patients: 299
- Number of Variables: 13
- Predictor Variables: 12
- Outcome Variable: DEATH_EVENT
- Missing Values: None
- Data Types: Integer, Continuous, and Binary

## Source

Chicco D, Jurman G. Machine learning can predict survival of patients with heart failure from serum creatinine and ejection fraction alone. BMC Medical Informatics and Decision Making. 2020;20:16. https://doi.org/10.1186/s12911-020-1023-5
